# Does physical exercise improve the capacity for independent living in people with dementia or mild cognitive impairment: an overview of systematic reviews and meta-analyses

**DOI:** 10.1080/13607863.2021.2019192

**Published:** 2021-12-24

**Authors:** Ahmet Begde, Manisha Jain, Eef Hogervorst, Thomas Wilcockson

**Affiliations:** School of Sport, Exercise and Health Sciences, Loughborough University, Loughborough, UK

**Keywords:** Exercise, activities of daily living, visual processing, mobility, dementia, mild cognitive impairment

## Abstract

**Objective:**

To summarise existing systematic reviews which assessed the effects of physical exercise on activities of daily living, walking, balance and visual processing in people with dementia or mild cognitive impairment

**Methods:**

In this overview of systematic reviews and meta-analyses, seven electronic databases were searched to identify eligible reviews published between January 2015 and April 2021.

**Results:**

A total of 30 systematic reviews were identified and included in the overview. The most frequent type of exercise for the intervention group was multimodal exercises. Mind-body exercises, exergames, dance intervention and aerobic exercise were other exercise types. Most of the reviews reported that exercise is significantly effective for improving activities of daily living (SMD 95%CI, from 0.27 to 1.44), walking (SMD 95%CI, from 0.08 to 2.23), balance (SMD 95%CI, from 0.37 to 2.24) and visuospatial function (SMD 95%CI, from 0.16 to 0.51), which are among the most leading determinants of independent living in individuals with dementia or mild cognitive impairment.

**Conclusion:**

Evidence has shown that exercise (especially multicomponent exercise programmes including cognitive, physical and multitasking exercises) with sufficient intensity improves the activities of daily living skills. Exercise also improves walking, balance and visual processing, which can provide a more independent life for people with dementia and mild cognitive impairment. Cognitively impaired people should therefore be encouraged to exercise regularly in order to be more independent.

Supplemental data for this article is available online at http://dx.doi.org/10.1080/13607863.2021.2019192

## Introduction

Worldwide, approximately 50 million people have dementia and this prevalence is expected to almost double by 2030 due to the aging global population (WHO, 2020). Dementia is one of the leading causes of mortality, morbidity and loss of functionality because of affected cognitive and physical ability (Laver et al., 2016; Todd et al., 2013). This functional decline can limit the performance of activities of daily living (ADLs) and lead to the need for assistance with daily activities, which has a negative impact on independence (Andersen et al., 2004). ADLs include basic daily activities such as eating, toileting, bathing and dressing, and instrumental ADLs (IADLs) such as banking, shopping and cooking require planning, memory and other higher cognitive functions (Lawton & Brody, 1969; Millán-Calenti et al., 2010). IADL and ADL are both essential care needs to live independently (Edemekong et al., 2017). Limitations in performing ADLs and IADLs are significantly exacerbated by dysfunctions in vision and visual perception, body balance control and mobility (Campos et al., 2019; Toots et al., 2016).

Previous studies have reported that impairments seen in both low-level and high-level visual processing negatively affect physical functions and ADL skills in people with dementia (Bowen et al., 2016; Ramzaoui et al., 2018). Jacobs et al. (2005) suggested that visually impaired people showed more significant dependence in both ADL and IADL. Problems in visual functions affect the independence of people with dementia either directly (Nyman et al., 2017) by affecting information processing or indirectly by impairing mobility and balance (Campos et al., 2019). Likewise, walking and balance problems in individuals with dementia adversely affect functional independence in performing (I)ADLs (Pitkälä et al., 2013). Although these dysfunctions can be seen to be mild in elderly individuals with or without mild cognitive impairment (MCI), they are often more severe in individuals with dementia (Hunter et al., 2020; Jutten et al., 2017). In addition, there is a positive correlation between the severity of these dysfunctions and the stage of dementia (Formiga et al., 2009). These factors (vision, gait and balance) can also affect falls risk, which is doubled in dementia, often leads to long-term hospitalisation, delirium and mortality, and worsens dementia outcomes (Härlein et al., 2009; Kearney et al., 2013).

Evidence suggests that exercise significantly reduces the risk of dementia in healthy older adults and people with MCI, which increases the risk of dementia (Alty et al., 2020). Similarly, regular exercise plays a critical role in delaying the progression from MCI to dementia (George & Reddy, 2019). Many reviews have been published examining the effect of exercise on dependence in ADL in people with dementia and MCI. Whereas some reviews have reported positive effects of exercise on ADLs (Forbes et al., 2015; Groot et al., 2016), others have found this effect statistically non-significant (Li et al., 2019; Zhao et al., 2020). These conflicting results might be due to the inclusion criteria of reviews, such as types of exercise, stage of the disease or the use of different assessment tools. A previous review of other systematic reviews (McDermott et al., 2019) of various psychosocial interventions including exercise found overall beneficial effects of these interventions on mood, cognition, ADL and gait speed, especially of multicomponent exercises including a cognitive component. This current systematic review of reviews seeks to extend and add to that paper with recently updated studies and includes visuospatial performance which can also impact falls risk (see above).

This overview investigates the effects of physical exercise on the performance of ADLs, visual processing, gait and balance, which are among the principal determinants of independent living in individuals with dementia. The present synthesis of reviews also examines which type, frequency, duration and intensity of exercise are more effective on these outcomes.

## Methods

The review protocol was registered in the PROSPERO database under the ID CRD42021247812. This review was carried out according to the guidelines of the Preferred Reporting Items for Systematic Reviews and Meta-Analyses (Moher et al., 2009) (see supplementary material 1).

### Eligibility criteria

To identify eligible reviews, the following inclusion criteria were defined: (1) systematic reviews and meta-analyses published in English after 2015, (2) population: patients with dementia and MCI, (3) intervention: any type of physical exercise or activity, and (4) outcomes: ADL, walking ability, balance and visual processing.

Reviews including the following criteria were excluded: (1) a healthy population, (2) the presence of other neurological diseases such as Parkinson and stroke, (3) interventions for caregivers, and (4) interventions for preventing dementia.

### Search strategy

To find eligible reviews, the following databases were searched: the Cochrane Central Register of Controlled Trials (CENTRAL) via the Cochrane, MEDLINE, EMBASE, PsycINFO, AMED, CINAHL and the Physiotherapy Evidence Database (PEDro). A comprehensive search strategy was created using the search terms of reviews published on similar topics and adapted to each database. The search strategy for MEDLINE is given as a supplementary material (2). In order to identify additional reviews, the reference lists of identified studies were also searched.

### Study selection

Irrelevant articles were excluded by screening the title and abstract of identified studies after excluding duplicate articles using a reference management software, EndNote X9. Then, to select appropriate studies for inclusion, the full texts of the remaining studies were scanned.

### Data collection

The data of the identified studies were independently extracted by two review authors (AB-MJ) using a data extraction tool covering the following information: author(s), year of publication, the number of studies included, population (the number of participants, age, gender), interventions (type and duration of treatment period) and outcome measures (type and assessment tools), and results (effect size 95%CI, heterogeneity, P-value). Disagreements were discussed with and resolved by a third reviewer.

### Assessment of methodological quality

The quality of the included reviews was assessed using the A Measurement Tool to Assess Systematic Reviews (AMSTAR2) tool. AMSTAR2 is a reliable and valid checklist consisting of 16 items for evaluating the quality of systematic reviews (Lorenz et al., 2019). AMSTAR 2 rates the confidence in the results of a review as critically low, low, medium or high. The quality assessment was independently carried out by the two review authors (AB-MJ). Disagreements were resolved by consensus with a third reviewer.

### Data management and statistical analysis

All data received from reviews were reported in table form and analysed narratively to summarise the results. When analysing the reviews descriptively, the numbers given to each review in Table 1 were used to refer to the reviews. Appropriate data were included in a forest plot to compare the effect sizes graphically using standardised mean differences (SMDs). We used data from meta-analyses conducted by authors. Additional meta-analyses were not performed. If the results were presented as mean differences, SMDs were calculated to be able to compare effect sizes across reviews. Additionally, considering that a primary study might be included in more than one review, the corrected covered area (CCA) method was used to determine the percentage of the overlapped primary studies in the included systematic reviews (Pieper et al., 2014). The primary studies included in each review were extracted into a table to calculate the overlapping percentage (*see* supplementary material 3).

**Table 1.** Characteristics of reviews.

Review	Number of studies	Participants	Interventions (length x frequency x duration)	Outcome measures	Effect sizes (95% Confidence Interval), I2 (%), P value
Almeida et al. (2020)	*n* = 16	Patients with dementia (*n* = 1129) Mean age: 77.3 (from 51 to 99) Gender: 49% female	Physical activities, strength, endurance and flexibility exercises (20 min–12 hr x 1–7 days/wk x 8–104 wk)	ADL (Katz Index, ADCS-ADL, BI, The 16-item self-reported assessment tool, LBS, IDDAD) Balance (FRT)	SM*D* = 0.80 (0.53, 1.07), I^2^ (%) =99, *P* = 0.00 SM*D* = 2.24 (1.80, 2.68), I^2^ (%) =97, *P* = 0.00
Brett et al. (2016)	*n* = 12	Patients with dementia (*n* = 901) Mean age: 82.6 Gender: NR	Multimodal exercises, walking, dance and hand exercises (30–120 min x 2–7 days/wk x 4–52 wk)	ADL (Katz Index of ADLs, BI, ACIF) Walking (6MWT, 2MWT, Locometer) Balance (BBS, One-leg balance test)	No meta-analysis
Cai et al. (2020)	*n* = 19	Patients with dementia and MCI (*n* = 1970) Mean age: 66 to 82 Gender: 68% female	Tai chi (20–120 min x 1–5 days/wk x 10–52 wk)	Visuospatial function: CDT, BDT	SM*D* = 0.03 (−0.28, 0.33), I^2^ (%) = 55, *P* = 0.7
Chan et al. (2020)	*n* = 5	Patients with MCI (*n* = 358) Mean age: from 67 to 76 Gender: 67% female	Dance interventions (25–60 min x 1–3 days/wk x 12–40 wk)	Visuospatial function: TMT	SM*D* = 0.16 (0.01, 0.32), I^2^ (%) = 0, *P* = 0.03
Farhang et al. (2019)	*n* = 9	Patients with MCI (*n* = 710) Mean age: 65 to 75 Gender: NR	Mind-body interventions, yoga and tai chi (30–90 min x 1–7 days/wk x 6–26 wk)	ADL: B-ADL-25 Walking: Speed Balance: BBS Visuospatial function: BDT, ANT	No meta-analysis
Forbes et al. (2015)	*n* = 16	Patients with dementia (*n* = 937) Mean age: 74.3 Gender:69% female	Cycling, walking, strength, aerobic, non-aerobic and combined physical activities (20–75 min x 1–5 days/wk x 2–78 wk)	ADL: Katz Index of ADLs, BI, CADS	SM*D* = 0.68 (0.08, 1.27), I^2^ (%) = 77, *P* = 0.03
Groot et al. (2016)	*n* = 18	Patients with dementia (*n* = 802) Mean age: 79.7 ± 4.2 Gender: 68% female	Aerobic, non-aerobic and combined physical activities (40–840 min/wk x 6–52 wk)	ADL: JTT, BI, IADL	SM*D* = 1.18 (0.57, 1.79), I^2^ (%) = NR, *P* < 0.01
Karssemeijer et al. (2017)	*n* = 10	Patients with dementia, AD and MCI (*n* = 742) Mean age: 72.1 Gender: 59% female	Combined cognitive and physical exercises (30–120 min x 2–6 days/wk x 8–52 wk)	ADL: DAD-ADL, E-ADL, ADCS-ADL, Bayer ADL	SM*D* = 0.65 (0.09, 1.21), I^2^ (%) = 80, *P* < 0.01
Lam et al. (2018)	*n* = 43	Patients with dementia and MCI (*n* = 3988) Mean age: 68 to 89 Gender: NR	Aerobic exercise, walking, dual-task walking, multimodal, stretching (30–150 min x 1–7 days/wk x 12–52 wk)	ADL: BI, Performance test of ADL, Katz ADL score, JTT Walking: 2MWT, 6MWT, step length, speed Balance BBS, TB, One-leg balance, FRT	M*D* = 9.59 (3.02, 16.16), I^2^ (%) = 89, *P* = 0.004 M*D* = 6MWT: 49.54 (17.70, 81.38), speed: 0.13 (0.03, 0.24), I^2^ (%) =6MWT: 79, speed: 90, *P* = 6MWT: 0.002, speed: 0.01 MD = BBS: 3.61 (0.26, 6.95), FRT: 3.85 (2.18, 5.53), I^2^ (%) = BBS: 91, FRT: 0, P = BBS: 0.03, FRT: 0.49
Lee et al. (2016)	*n* = 9	Patients with dementia (*n* = 228) Mean age: NR Gender: NR	Dance therapy, tai chi, strength and balance exercises, walking (30–150 min x 1–7 days/wk x 12–52 wk)	ADL: BI, ADL, IADL	SM*D* = 0.73 (0.23, 1.23), I^2^ (%) = NR, *P* = 0.004
Leng et al. (2018)	*n* = 21	Patients with dementia, AD and MCI (*n* = 2589) Mean age: 67 to 89 Gender: 64% female	Resistance training, yoga, tai chi, cycling, walking, strength, balance and aerobic exercises (15–90 min x 1–7 days/wk x 6–64 wk)	ADL: BI, Katz Index of ADLs, ADCS-ADL, CDAD-IADL	SM*D* = 0.27 (0.12, 0.43), I^2^ (%) = 0, *P* = 0.0005
Lewis et al. (2017)	*n* = 7	Patients with dementia (*n* = 945) Mean age: 74 to 82 Gender: 64% female	Long-term multicomponent exercise, treadmill walking, chair-based exercise (15–90 min x 1–7 days/wk x 16–52 wk)	ADL: ADL, IADL, FIM Balance: SPPB, BBS, FRT	SMD = ADL: 0.77 (0.17, 1.37), IADL: 0.44 (0.03, 0.86), I^2^ (%) = ADL: 67, IADL: 42, P = ADL: 0.01, IADL: 0.04 M*D* = 5.2 (0.5, 9.9), I^2^ (%) = 76, *P* = 0.03
Li et al. (2019)	*n* = 20	Patients with dementia (*n* = 2051) Mean age: 70.5 to 87.9 Gender: 60% female	Strength and balance exercises including walking, squats, and trunk exercises, walking (6–52 wks)	ADL: Bristol ADL, ADCS-ADL, BI, FIM, IADL, CADS	SM*D* = 0.50 (−0.03, 1.02), I^2^ (%) = 95, *P* = 0.066
Lim et al. (2019)	*n* = 9	Patients with dementia (*n* = 656) Mean age: 78 Gender: NR	Tai chi chuan (20–60 min x 1–4 days/wk x 8–52 wk)	Visuospatial function: BDT	No meta-analysis
Long et al. (2019)	*n* = 8	Patients with moderate-to-severe dementia (*n* = 819) Mean age: ≥65 Gender: NR	Cycling, multicomponent exercises, walking (15–60 min x 2–7 days/wk x 15–65 wk)	Walking: 2MWT, 6MWT, 10MWT, TUG	No meta-analysis
Machado et al. (2020)	*n* = 6	Patients with dementia (*n* = 489) Mean age: 80.9 (from 51 to 93) Gender: 67% female	Multicomponent exercise (30–60 min x 1–2 days/wk x 4–52 wk)	ADL: BI, IADL, ADCS-ADL, FIM, Katz ADL Walking: Speed Balance: SPPB, One-leg balance test	SM*D* = 0.31 (0.16, 0.46), I^2^ (%) = 8, *P* < 0.01 No meta-analysis No meta-analysis
Marques et al. (2019)	*n* = 2	Patients with AD (*n* = 207) Mean age: 65 to 100 Gender: NR	Aerobic or anaerobic exercises (30 min x 3–5 days/wk x 26–52 wk)	ADL: Pfeffer instrumental activities questionnaire	No meta-analysis
Russ et al. (2020)	*n* = 9	Patients with dementia (*n* = 456) Mean age: 85.5 Gender: 74.1% female	High-intensity functional exercise (45–60 min x 2–3 days/wk x 12–16 wk)	ADL: BI, FIM Walking: speed Balance: BBS	SM*D* = 0.30 (0.11, 0.49), I^2^ (%) =0, *P* = 0.002 No meta-analysis M*D* = 2.30 (0.44, 4.16), I^2^ (%) =73 *P* = 0.02
Sultana et al. (2020)	*n* = 5	Patients with dementia, AD and MCI (*n* = 150) Mean age: from 65 to 85 Gender: NR	Exergames Wii Fit^©^ Time, duration and frequency: NR	Balance: BBS, TUG	SM*D* = 0.46 (0.08, 0.84), I^2^ (%) = 0, *P* = 0.02
Van Santen et al. (2018)	*n* = 3	Patients with dementia (*n* = 71) Mean age: 75 Gender: 59% female	Exergames (30–60 min x 3–5 days/wk x 7–8 wk)	ADL: ADL, IADL Walking: TUG Balance: BBS, Tinetti	No meta-analysis
Wei et al. (2020)	*n* = 12	Patients with dementia (*n* = 981) Mean age: 60 to 85 Gender: NR	Tai chi (20–60 min x 2–5 days/wk x 8–26 wk)	ADL: Lawton’s IADL, FAQ Visuospatial Function: BDT VST	No meta-analysis
Yang et al. (2020)	*n* = 11	Patients with MCI (*n* = 1061) Mean age: 74.1 Gender: 71% female	Tai chi (30–120 min x 1–6 days/wk x 10–52 wk)	Visuospatial function: RFT, BDT, BCT	SM*D* = 0.29 (0.10, 0.48), I^2^ (%) = 0, *P* = 0.003
Yeh et al. (2021)	*n* = 15	Patients with dementia (*n* = 860) Mean age: ≥65 Gender: 72% female	High-intensity functional exercise (45–120 min x 2–3 days/wk x 12–16 wk)	ADL: BI Walking: 4MWT, 6MWT, GAITRite Balance: BBS, inertial sensor	SM*D* = 0.80 (0.23, 1.38), I^2^ (%) =0, *P* = 0.006 SMD: 0.08 (−0.02, 0.18), I^2^ (%) =75, *P* = 0.04 SMD: 0.57 (0.31, 0.83), I^2^ (%) =54, *P* = 0.0001
Zhang et al. (2019)	*n* = 5	Patients with dementia, AD and MCI (*n* = 803) Mean age: 65 to 79 Gender: NR	Traditional Chinese exercises including tai chi, baduanjin, qigong, Jinjiang, liuzijue and wuqinxi (20–50 min x 3–5 days/wk x 12–52 wk)	Visuospatial function: VS, BDT	SM*D* = 0.38 (0.22, 0.54), I^2^ (%) = 0, *P*<.001
Zhao et al. (2020)	*n* = 10	Patients with dementia and MCI (*n* = 702) Mean age: 79.8 Gender: 58% female	Exergaming training and virtual reality-based exercises (30–120 min x 1–5 days/wk x 4–24 wk	ADL: BI, IADL Balance: BBS, wearable sensors Visuospatial function: ROCFT	No meta-analysis
Zheng et al. (2016)	*n* = 11	Patients with dementia (*n* = 1497) Mean age: 74.1 Gender: 62% female	Aerobic exercises (30–60 min x 2–5 days/wk x 26–52 wk)	Visuospatial function: UFOV	No meta-analysis
Zhou et al. (2020)	*n* = 11	Patients with MCI (*n* = 676) Mean age: from 68 to 79 Gender: 66% female	Multicomponent exercise, resistance exercise, aerobic exercise, high-speed elastic band training, tai chi, yoga (12–52 wk)	Visuospatial function: BDT	SM*D* = 0.38 (0.03, 0.72), I^2^ (%) = 0, *P* = 0.03
Zhu et al. (2015)	*n* = 23	Patients with AD (*n* = 886) Mean age: from 50 to 96 Gender: 66% female	Aerobic, tai chi, dance, walking, physical activities (6–52 wk)	ADL: BI, IADL Walking: 6MWT, Speed, TUG Balance: BBS	SM*D* = 0.78 (0.33, 1.23), I^2^ (%) = 62, *P* = 0.0007 SM*D* = 6MWT: 2.23 (−0.47, 4.93), speed: 0.16 (−0.14, 0.47), I^2^ (%) = 6MWT: 96, speed: 19, *P* = 6MWT: 0.11, speed: 0.29 SM*D* = 1.11 (0.37, 1.84), I^2^ (%) = 66, *P* = 0.003
Zhu et al. (2020)	*n* = 16	Patients with AD (*n* = 1181) Mean age: 69 to 83 Gender: NR	Aerobic, strength, walking, physical activities, flexibility (30–150 min x 1–7 days/wk x 8–48 wk)	ADL: BI, IADL, Physical function of SF36, ADCS-ADL	SM*D* = 0.68 (0.19, 1.16), I^2^ (%) = 86, *P* < 0.05
Zou et al. (2019)	*n* = 12	Patients with MCI (*n* = 1298) Mean age: from 60 to 77.8 Gender: 68% female	Tai chi, yoga, qigong (30–90 min x 1–6 days/wk x 12–52 wk)	Visuospatial function: BDT, Copy cube/ TMT b	SM*D* = 0.51 (0.15, 0.87), I^2^ (%) = 0, *P* = 0.01

2MWT, 2-minute walk test; 6MWT, 6-minute walk test; 8MWT, 8-minute walk test; 10MWT, 10-minute walk test; ACIF, Acute Care Index of Function; AD, Alzheimer’s disease; ADCS-ADL, Alzheimer’s Disease Cooperative Study Group Activities of Daily Living scale; ADL, activities of daily living; ANT, Animal Naming Test; B-ADL-25, Bayer Activities of Daily Living; BBS, Berg balance scale; BDS, block design score; BI, Barthel Index; CADS, Changes in Advanced Dementia Scale; CDT, Clock-Drawing Test; DAD-ADL, Disability Assessment for Dementia subscale Activities of Daily Living; E-ADL, Erlangen test of Activities of Daily Living; FRT, Functional Reach Test; GS, gait speed; JTT, Jebsen Taylor Hand Function Test; IADL, instrumental activities of daily living; IDDAD, Interview for Deterioration of Daily Activities in Dementia; LBS, Lawton and Brody’s scale; VS, visual span; UPDRS-II, Unified Parkinson’s disease rating scale subscale II (ADL); ROCFT, Rey-Osterrieth Complex Figure Test; SPPB, short physical performance battery; TUG, time and up go; UFOV, useful field of view.

## Results

**Figure 1.**
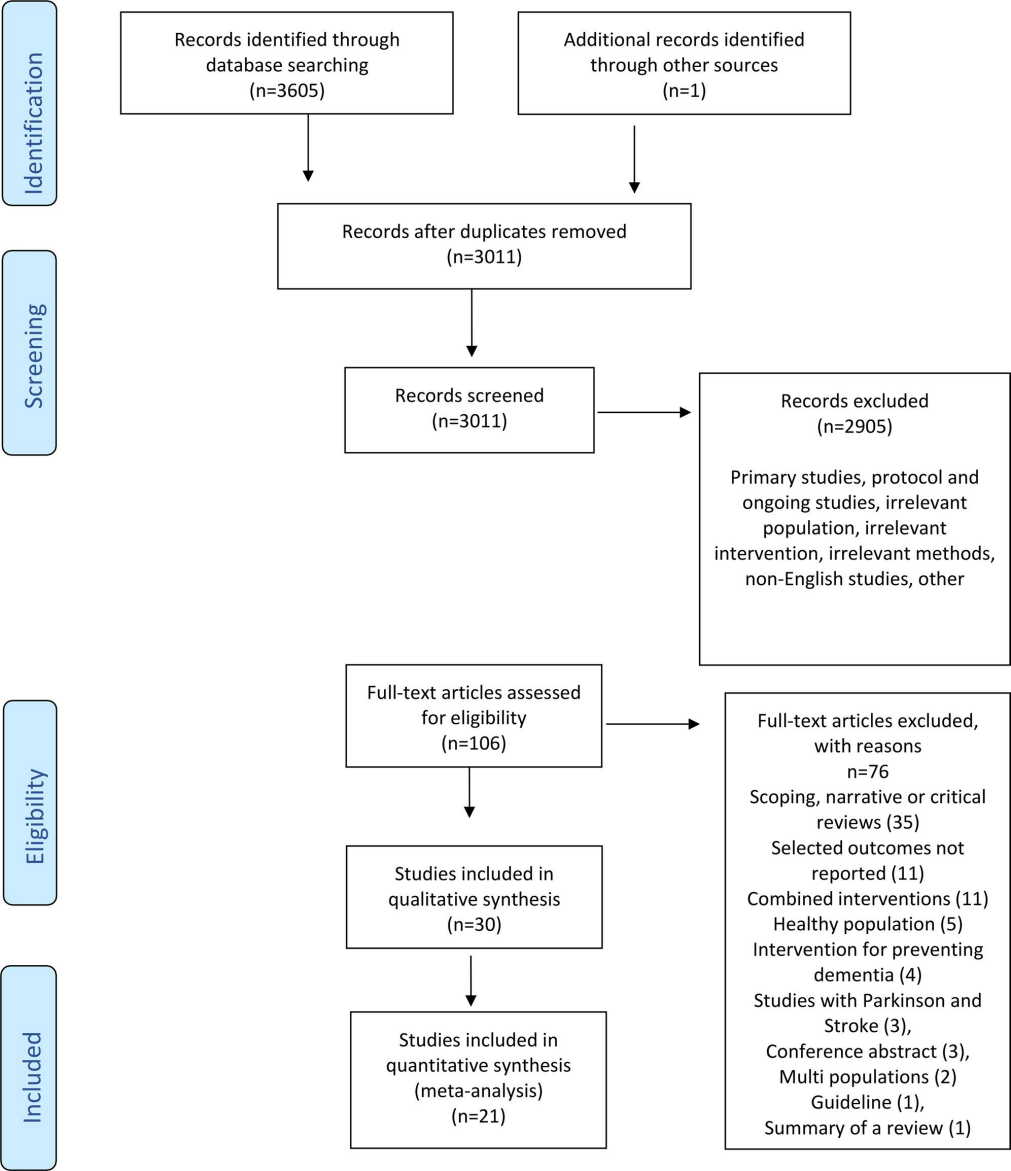
The PRISMA flow diagram.

The database searching and study selection processes are shown in Figure 1. A total of 3606 studies were identified by the initial electronic database search. Duplicates were removed using a reference manager software, EndNote X9. After scanning the titles and abstracts of the remaining 3011 studies, 2905 studies were determined to be irrelevant. The full text of 106 studies was reviewed, and a total of 30 reviews which met the inclusion criteria were identified and included in this overview.

### Characteristics of the included reviews

The characteristics of the included reviews are demonstrated in Table 1. Detailed data extraction table is provided as a supplementary material (4). The number of studies included in the reviews ranged from 2 to 43. All versions of dementia were included in 15 reviews (50%),^(1,2,6,7,10,12–^16,18,20,21,23,26^)^ whilst three reviews (10%)^(^17^,^28^,^29^)^ included only individuals with Alzheimer’s as a population. Five reviews (17%)^(^4^,^5^,^22^,^27^,^30^)^ included only people with MCI, whereas seven reviews (23%)^(^3^,^8^,^9^,^11^,^19^,^24^,^25^)^ included people with MCI and dementia.

The age of participants ranged from 50 to 100 and the mean age of the participants in the 27 reviews (90%)^(^1–9,11–15,17–27,29,30^)^ was over 60. Moreover, the mean age was greater than 70 in more than half of the reviews (53%).^(^1,2,6–8,12–14,16,18,20,22,25–27,29^)^ A total of 19 reviews^(^1,3,4,6–8,11–13,16,18,20,22,23,25–28,30^)^ reported gender proportions. Only Almeida et al. (2020) reported that the percentage of men (51%) was slightly higher than that of women (49%), whereas the percentage of women was much higher in the remaining 18 reviews (ranging from 58 to 74).

Most of the reviews (53%)^(^1,2,6,8–13,15,16,18,23,27–29^)^ examined the effects of different exercises or multimodal exercises as an intervention. Seven reviews (24%)^(^3^,^5^,^14^,^21^,^22^,^24^,^30^)^ specifically investigated the effect of mind-body exercises, such as tai-chi and yoga. Three reviews (10%)^(^19^,^20^,^25^)^ examined the effect of exergames. Likewise, the effectiveness of aerobic exercises was examined in three reviews (10%).^(^7^,^17^,^26^)^ Only one review (3%)^(^4^)^ investigated the therapeutic effect of dance intervention. Although the length of the exercise varied considerably across the reviews (ranging from 15 min to 150 min), exercise sessions of 30–60 min were commonly used. Similarly, the frequency (one to seven days) and duration (two weeks to two years) of exercise varied widely. In most of the reviews^(18),(^1–4,6–8,12,13,15–18,20,21,23,27,30^)^ treatments without physical exercises such as social activities, group reading, health education and music therapy were provided to the control group, whilst physical activities such as stretching, walking and exercise were among the treatments provided to the control group in nine reviews.^(^5,11,14,19,22,24–26,29^)^ The remaining three reviews^(^9^,^10^,^28^)^ did not provide information about control group interventions.

Most of the reviews (20)^(^1,2,5–13,16–18,20,21,23,25,28,29^)^ assessed ADL and 20 different assessment tools were used to assess the performance of ADLs. The most used tools were the Barthel Index (13), the Instrumental Activities of Daily Living scale (8), and the Alzheimer’s Disease Cooperative Study Group Activities of Daily Living scale (6). Among the seven different assessment methods in nine reviews^(^2^,^5^,^9^,^15^,^16^,^18^,^20^,^23^,^28^)^ assessing walking, the six-minute walk test (5) was the most used tool. Likewise, eight different tools were used to measure balance in 12 reviews.^(^1,2,5,9,12,16,18–20,23,25,28^)^ The Berg Balance Scale (10) was the most frequently used tool to measure balance. Among the five different tools used to assess the visuospatial function in 11 reviews,^(^3–5,14,21,22,24–27,30^)^ the Block Design Test (8) was also the most used tool.

### Quality assessment of the included reviews

As a result of the quality assessment with the 16-item AMSTAR 2 tool, only one Cochrane review^(^6^)^ was found to be a high-quality review (*see* supplementary material 5). Of the remaining 29 reviews, eight^(^4,5,8,15,20,25–27^)^ were interpreted as low quality and 21^(^1–3,7,9–14,16–19,21–24,28–30^)^ were critically low quality. The protocol of 12 reviews^(^1,4,6,8,15,16,20,21,23,25–27^)^ was registered (item 2). All reviews used a comprehensive search strategy (item 4). Only three reviews^(^5^,^617^)^ provided the list of excluded studies (item 7). In all reviews except one,^(^10^)^ the risk of bias was evaluated with a satisfactory technique (item 9). Of the reviews which performed a meta-analysis, only two^(^21^,^23^)^ did not provide sufficient information about the statistical combination of results (item 11). Six reviews^(^3^,^10^,^11^,^14^,^16^,^28^)^ did not interpret the effect of risk of bias on the results (item 13). Ten reviews^(^1,11,17–19,21–24,27^)^ did not evaluate publication bias (item 15).

### Data synthesis

#### Activities of daily living

**Figure 2.**
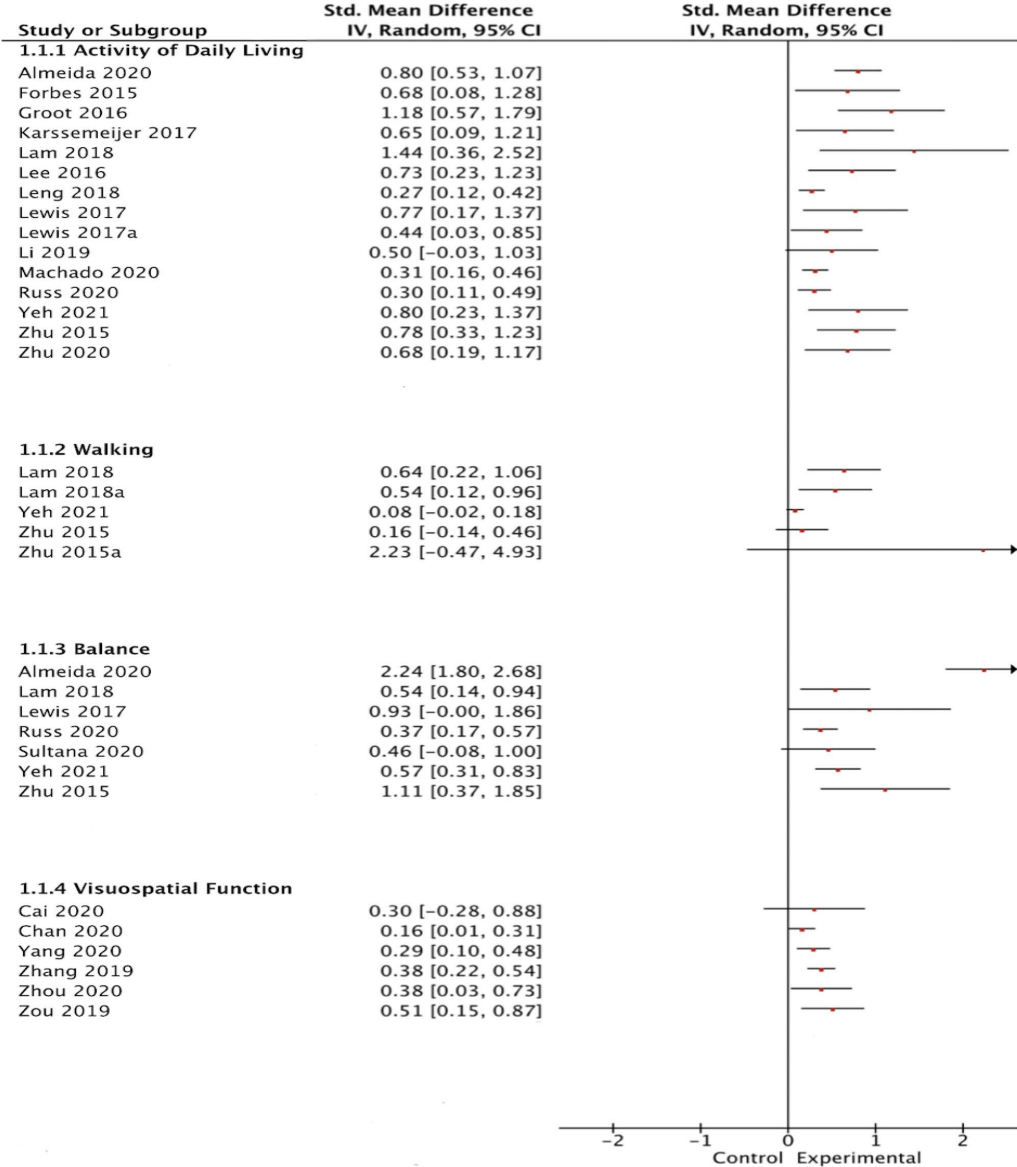
The forest plot comparing SMDs to show the efficacy of exercise on (I)ADL, walking, balance, and visuospatial function.

Of the 20 reviews using ADL as an outcome measure, 14 pooled the data of the primary studies and estimated that the effect size ranged from 0.27 to 1.44 (see Figure 2). Only one study reported that the positive effect of exercise was not superior to the control intervention (Li et al., 2019). Contradictory results were also found in the other six reviews^(^2^,^5^,^17^,^20^,^21^,^25^)^ in which meta-analysis was not performed. Although four of the six reviews^(^2^,^5^,^20^,^21^)^ reported that exercise significantly increased ADL, Marques et al. (2019), who examined aerobic and non-aerobic exercises, and Zhao et al. (2020), who examined the effect of exergames, reported that exercise had no significant effect compared with control interventions.

In 20 reviews with 59 different primary studies, the percentage of overlapping calculated by the CCA method was 4.8%. The reviews showed that multicomponent functional exercises or combined cognitive and physical exercises significantly improved ADL skills (Karssemeijer et al., 2017; Machado et al., 2020).

#### Walking

Three reviews evaluated the effect of exercise on walking and estimated effect sizes (from 0.08 to 2.23) by performing a meta-analysis (see Figure 2). Zhu et al. (2015) found that aerobic exercises such as tai-chi, yoga and walking were no more effective than control intervention in improving both walking speed and distance. The other two meta-analyses showed that functional exercises were significantly more effective in improving walking ability (Lam et al., 2018; Yeh et al., 2021).

Although most other reviews without meta-analysis^(^2^,^15^,^16^)^ reported positive effects of exercise, low-quality studies and contradictory results precluded achieving robust evidence. In eight reviews with a total of 26 primary studies, the percentage of overlapping for walking was 6.2%.

#### Balance

In six reviews, meta-analysis was performed to pool data from primary studies examining the effectiveness of exercise on balance (see Figure 2). All the meta-analysis results showed that exercise was more effective in improving balance than control interventions. Despite contradictory results in other reviews, most reviews^(^2^,^20^,^25^)^ reported a positive effect of exercise on balance. Across reviews, including 27 primary studies, the percentage of overlapping was 4.4%. The results showed that exergames, functional exercise and mind-body exercises were effective in improving balance (Lewis et al., 2017; Sultana et al., 2020).

#### Visuospatial function

Five reviews which calculated the effect size by meta-analysis found that visuospatial functions could significantly improve with exercise rather than with control interventions (see Figure 2). Only two reviews in which visuospatial functions were used as an outcome measure reported that exercise did not have a significantly positive effect (Cai et al., 2020; Wei et al., 2020). Moderate overlapping (6%) was also found for visuospatial function investigated in 11 reviews with 26 primary studies.

The visuospatial function was assessed in all five reviews^(^4^,^5^,^22^,^27^,^30^)^ that included only individuals with MCI. All these reviews reported that exercise training (dance, mind-body, and multimodal) significantly improved visuospatial functions. Only one review^(^5^)^ specifically included people with MCI and investigated the effectiveness of exercise on ADL, walking and balance. The review found that mind-body exercises did not significantly improve balance and walking ability whereas it has a significant impact on ADL.

## Discussion

This overview of systematic reviews and meta-analyses provides a synthesis of the best evidence on exercise for independent living in people with dementia and MCI. Our study synthesised 30 reviews containing the data of 88 primary studies and showed that exercise had significant positive effects on independent living for people with dementia and MCI.

Most reviews found that exercise significantly improved ADL performance. Only two reviews reported that exercise therapy did not have a significantly positive effect on ADL (Marques et al., 2019; Zhao et al., 2020). These two critically-low-quality reviews examined specific types of exercise, such as exergames and aerobic exercise. Marques et al. (2019) included only one study which had used ADL as an outcome measure, which indicates that the results should be interpreted according to the evidence of a single study rather than a review. Likewise, Zhao et al. (2020) reported that exergames did not significantly improve ADL skills. However, the authors also stated that this result was not a strong level of evidence and that further research is needed.

Reviews showed that 45–60 min x 3–5 days/week x at least a 12-week multimodal exercise programme or a combination of a cognitive and physical training programme can significantly improve ADL. Our results were consistent with those of previous overviews. In an overview of reviews, the effect of psychosocial treatments on individuals with dementia was examined (McDermott et al., 2019). Of the included reviews, only three reviews published before 2015 investigated the effect of exercise on the ADL skill. Although the included reviews examined a limited type of exercise, this overview reported that exercise significantly improves ADL and may be more effective than other treatments (cognitive training and cognitive rehabilitation).

In a more recent similar overview, Meyer and O’Keefe (2020) examined the effect of non-pharmacological treatments in individuals with dementia. In their overview, which included 38 reviews, six reviews had investigated the effectiveness of the exercise. The review reported that exercise showed the most robust evidence for improving ADL. Another umbrella review published in 2020 specifically examined the effectiveness of physical activity and exercise on both cognitive and non-cognitive outcomes (Demurtas et al., 2020). Seven of the included reviews were conducted with ADL as an outcome measure and only one review found no positive results (Bruderer-Hofstetter et al., 2018). However, this included study had investigated the efficacy of multicomponent treatments alongside exercise rather than exercise only, which could have undermined the validity of the result.

Our overview found the least level of evidence showing the effectiveness of exercise for walking outcomes. Only a few of the reviews achieved robust results reporting positive effects of exercise (Lam et al., 2018; Yeh et al., 2021) whilst others reported contradictory results (Brett et al., 2016; Long et al., 2019; Zhu et al., 2015). Moreover, the review investigating the effect of exercise on walking ability was limited, and the percentage of overlap between these reviews was higher than other outcomes.

Interestingly, reviews showed that, contrary to positive developments in other outcomes, exergames and multicomponent exercises were not more effective than control interventions in improving walking (Machado et al., 2020; Van Santen et al., 2018). However, the duration of treatment in studies reporting this finding was shorter (30 min) than recommended (45–60 min), suggesting that these doubtful results could result from poorly designed studies. Our review showed that high-intensity (at least 60 min) functional exercise seemed more effective in improving walking ability.

A recent review of reviews examining the effects of exercise on physical and cognitive functions in individuals with dementia and their caregivers reported findings consistent with our results (Lewis et al., 2020). Although the types and years of included reviews differed from our inclusion criteria, exercise was reported to significantly improve walking in individuals with dementia. Likewise, the synthesis of systematic reviews carried out by McDermott et al. (2019) showed that exercise could be an effective psychological intervention in improving walking speed.

Our review found the highest level of evidence for the balance outcome, in which the primary studies included had the lowest overlapping rate. Reviews reported that exercise significantly improved balance in individuals with dementia. Although all the included meta-analyses showed that exercise was statistically more effective than control treatments in improving balance, some reviews (without meta-analysis) found contradictory results (Brett et al., 2016; Farhang et al., 2019). Two studies examining the effect of multimodal exercises in the review by Brett et al. (2016) found different results (Christofoletti et al., 2008; Rolland et al., 2007**)**. These differing results may also be due to the intensity of exercise. In Christofoletti’s study, multimodal exercises were provided on 120-min sessions five days a week and significant results were found, whereas Rolland provided a 60-min exercise programme two days a week and achieved statistically insignificant results. Another review conducted by Farhang et al. (2019) also found that mind-body exercises were ineffective in improving balance.

The results of the included reviews showed that exergames and high-intensity functional exercises (at least 60 min x 5 days/week) were effective in improving balance. Similar results were found in a recent overview examining the effect of physical exercises combined with cognitive exercises in cognitively impaired people (Gallou-Guyot et al., 2020). Although the reviews included were low and critically low quality, such exercise programmes were reported to significantly improve balance and gait.

As in other outcomes, the effectiveness of many different types of exercise was investigated for visuospatial function. Reviews showed that exercise training could significantly improve visuospatial function in individuals with dementia. In particular, dance therapy, exergames and multicomponent exercises were significantly effective in improving visuospatial function. Mind-body and traditional Chinese exercises were also found to be effective, although the effectiveness of tai-chi was not demonstrated with solid evidence. Conflicting findings were reported on the effectiveness of tai-chi. Although a meta-analysis conducted by Cai et al. (2020) reported that tai-chi was not significantly effective in improving visuospatial functions, another meta-analysis found statistically significant results in favour of tai-chi (Yang et al., 2020). Interestingly, although both meta-analyses were performed with data from the same studies, statistically opposite results were found. The fact that one of the reviews did not publish the meta-analysis details or a forest plot prevents us from interpreting what caused this difference. However, the results of other reviews (Farhang et al., 2019; Lim et al., 2019) in which a meta-analysis was not performed supported the results reported by Yang et al. (2020).

### Strengths and limitations

This overview of reviews allows a comprehensive synthesis of the results of SRs which are at the top of the evidence hierarchy and provide high-evidence-level results. Although this overview provides robust results by synthesising high-quality and recent evidence from different types of exercise and combining the best available evidence, the unique limitations of this approach should be mentioned. First, the high heterogeneity between reviews resulting from different exercise types, disease stages and outcome measures makes it challenging to synthesise evidence effectively and easily. Second, most reviews gave exercise intensity as a range rather than a mean value, making it difficult to determine optimal exercise intensity parameters. Third, the inclusion of some primary studies in more than one review and counting some data more than once might cause bias by overestimating the effect of the treatment, although the overlapping rate in the included reviews was calculated to be between 4.2% and 6.8%. Therefore, instead of conducting a network meta-analysis of the results of the reviews, it is recommended to re-analyse the data of the primary studies to determine the most effective treatment method (Pollock et al., 2018). However, we only compared the effect-sizes found as a result of the meta-analysis without performing an additional meta-analysis. This prevents us from statistically comparing exercise methods and reaching the most effective exercise type.

Another limitation of the review is that other outcomes such as cognitive functions and muscle strength which could affect the independence of individuals in ADL are not included in the review. Finally, we did not include falls risk. However, a review of reviews examining the effects of different types of interventions on falls risk in individuals with dementia showed the positive impacts of exercise on the risk of falling (Booth et al., 2015).

### Implications for future research and practice

Our review showed that exercise therapy could increase the independence of individuals with dementia by improving outcomes closely related to ADL and IADL. This can be very effective in reducing healthcare costs. Moreover, because exercise therapy might be more cost-effective than usual care and other treatments (Laver et al., 2016), policy-makers and practitioners should consider the findings of this review, which can reduce the high costs of dementia.

Although there are many reviews in the literature examining the effect of exercise, most of them were critically low quality (see supplementary material 5). High-quality systematic reviews should therefore be conducted to examine the effect of exercise in individuals with dementia. Additionally, we did not find any network meta-analyses which had examined physical outcomes such as ADL, balance and walking. A recently published network meta-analysis compared exercise types (Huang et al., 2021) but in that review, in which ADL was the secondary outcome, network analysis was performed only for primary outcomes; therefore, a high-quality network meta-analysis comparing the effectiveness of different exercise types should be carried out.

Multicomponent exercise, which seems more effective in improving ADL, balance and visuospatial functions in individuals with dementia, is a broad definition which includes different types of exercise. Thus, the content of the exercise programme (in terms of type and intensity) should be outlined in detail with high-quality RCTs. Likewise, the disease (its stage/type) should be examined more specifically in studies and reviews. The limited number of reviews that included only individuals with MCI prevented us from obtaining robust results showing the effect of exercise on this population. Therefore, there is a need for reviews and studies based on the type and level of the disease.

## Conclusion

In conclusion, this review found that exercise therapy can significantly improve ADL, walking, balance, visuospatial functions which are crucially important for independent living in individuals with dementia. The results showed that providing a multicomponent exercise programme with moderate intensity, including cognitive, physical and multitasking exercises, might be more effective than other types of exercise. However, since the methodological quality of most of the reviews published so far to explore this topic has been critically low, the results should be carefully interpreted and adapted to clinical practice. In future reviews, the content of the multicomponent exercise programme should be investigated and detailed using high-quality studies.

## Supplementary Material

Supplemental Material

## References

[CIT0001] Almeida, S. I. L., Gomes da Silva, M., & Marques, A. S. P. d D. (2020). Home-based physical activity programs for people with dementia: Systematic review and meta-analysis. *The Gerontologist*, *60*(8), 600–e608. 10.1093/geront/gnz176 31858111

[CIT0002] Alty, J., Farrow, M., & Lawler, K. (2020). Exercise and dementia prevention. *Practical Neurology*, *20*(3), 234–240. 31964800 10.1136/practneurol-2019-002335

[CIT0003] Andersen, C. K., Wittrup-Jensen, K. U., Lolk, A., Andersen, K., & Kragh-Sørensen, P. (2004). Ability to perform activities of daily living is the main factor affecting quality of life in patients with dementia. *Health and Quality of Life Outcomes*, *2*(1), 52–57. 10.1186/1477-7525-2-52 15383148 PMC521495

[CIT0004] Booth, V., Logan, P., Harwood, R., & Hood, V. (2015). Falls prevention interventions in older adults with cognitive impairment: A systematic review of reviews. *International Journal of Therapy and Rehabilitation*, *22*(6), 289–296. 10.12968/ijtr.2015.22.6.289

[CIT0005] Bowen, M., Edgar, D. F., Hancock, B., Haque, S., Shah, R., Buchanan, S., Iliffe, S., Maskell, S., Pickett, J., & Taylor, J.-P. (2016). The Prevalence of Visual Impairment in People with Dementia (the PrOVIDe study): A cross-sectional study of people aged 60–89 years with dementia and qualitative exploration of individual, carer and professional perspectives. 10.3310/hsdr04210 27489923

[CIT0006] Brett, L., Traynor, V., & Stapley, P. (2016). Effects of physical exercise on health and well-being of individuals living with a dementia in nursing homes: A systematic review. *Journal of the American Medical Directors Association*, *17*(2), 104–116. 26432622 10.1016/j.jamda.2015.08.016

[CIT0007] Bruderer-Hofstetter, M., Rausch-Osthoff, A.-K., Meichtry, A., Münzer, T., & Niedermann, K. (2018). Effective multicomponent interventions in comparison to active control and no interventions on physical capacity, cognitive function and instrumental activities of daily living in elderly people with and without mild impaired cognition—A systematic review and network meta-analysis. *Ageing Research Reviews*, *45*, 1–14. 10.1016/j.arr.2018.04.002 29679658

[CIT0008] Cai, Z., Jiang, W., Yin, J., Chen, Z., Wang, J., & Wang, X. (2020). Effects of Tai Chi Chuan on cognitive function in older adults with cognitive impairment: A systematic and meta-analytic review. *Evidence-Based Complementary and Alternative Medicine*, *2020*, 1–11. 10.1155/2020/6683302 PMC7781704 33424991

[CIT0009] Campos, J. L., Höbler, F., Bitton, E., Labreche, T., McGilton, K. S., & Wittich, W. (2019). Screening for vision impairments in individuals with dementia living in long-term care: A scoping review. *Journal of Alzheimer’s Disease: JAD*, *68*(3), 1039–1049. 10.3233/JAD-181129 PMC6484267 30909236

[CIT0010] Chan, J. S., Wu, J., Deng, K., & Yan, J. H. (2020). The effectiveness of dance interventions on cognition in patients with mild cognitive impairment: A meta-analysis of randomized controlled trials. *Neuroscience and Biobehavioral Reviews*, *118*, 80–88. 10.1016/j.neubiorev.2020.07.017 32687886

[CIT0500] Christofoletti, G., Oliani, M. M., Gobbi, S., Stella, F., Bucken Gobbi, L. T., & Renato Canineu, P. (2008). A controlled clinical trial on the effects of motor intervention on balance and cognition in institutionalized elderly patients with dementia. *Clinical Rehabilitation*, *22*(7), 618–626. 18586813 10.1177/0269215507086239

[CIT0011] Demurtas, J., Schoene, D., Torbahn, G., Marengoni, A., Grande, G., Zou, L., Petrovic, M., Maggi, S., Cesari, M., Lamb, S., Soysal, P., Kemmler, W., Sieber, C., Mueller, C., Shenkin, S. D., Schwingshackl, L., Smith, L., & Veronese, N. (2020). Physical activity and exercise in mild cognitive impairment and dementia: An umbrella review of intervention and observational studies. *Journal of the American Medical Directors Association*, *21*(10), 1415–1422. e1416. 10.1016/j.jamda.2020.08.031 32981668

[CIT0012] Edemekong, P. F., Bomgaars, D. L., & Levy, S. B. (2017). Activities of daily living (ADLs). 29261878

[CIT0013] Farhang, M., Miranda-Castillo, C., Rubio, M., & Furtado, G. (2019). Impact of mind-body interventions in older adults with mild cognitive impairment: A systematic review. *International Psychogeriatrics*, *31*(5), 643–666. 30712518 10.1017/S1041610218002302

[CIT0014] Forbes, D., Forbes, S. C., Blake, C. M., Thiessen, E. J., & Forbes, S. (2015). Exercise programs for people with dementia. *Cochrane Database of Systematic Reviews*, *4*. 1–74. 10.1002/14651858.CD006489.pub4 PMC9426996 25874613

[CIT0015] Formiga, F., Fort, I., Robles, M., Riu, S., Sabartes, O., Barranco, E., & Catena, J. (2009). Comorbidity and clinical features in elderly patients with dementia: Differences according to dementia severity. *The Journal of Nutrition, Health & Aging*, *13*(5), 423–427. 10.1007/s12603-009-0078-x19390748

[CIT0016] Gallou-Guyot, M., Mandigout, S., Combourieu-Donnezan, L., Bherer, L., & Perrochon, A. (2020). Cognitive and physical impact of cognitive-motor dual-task training in cognitively impaired older adults: An overview. *Neurophysiologie Clinique = Clinical Neurophysiology*, *50*(6), 441–453. 10.1016/j.neucli.2020.10.010 33121880

[CIT0017] George, E. K., & Reddy, P. H. (2019). Can healthy diets, regular exercise, and better lifestyle delay the progression of dementia in elderly individuals? *Journal of Alzheimer’s Disease: JAD*, *72*(s1), S37–S58. 31227652 10.3233/JAD-190232

[CIT0018] Groot, C., Hooghiemstra, A., Raijmakers, P., Van Berckel, B., Scheltens, P., Scherder, E., Van der Flier, W., & Ossenkoppele, R. (2016). The effect of physical activity on cognitive function in patients with dementia: A meta-analysis of randomized control trials. *Ageing Research Reviews*, *25*, 13–23. 10.1016/j.arr.2015.11.005 26607411

[CIT0019] Härlein, J., Dassen, T., Halfens, R. J., & Heinze, C. (2009). Fall risk factors in older people with dementia or cognitive impairment: A systematic review. *Journal of Advanced Nursing*, *65*(5), 922–933. 10.1111/j.1365-2648.2008.04950.x 19291191

[CIT0020] Huang, X., Zhao, X., Li, B., Cai, Y., Zhang, S., Wan, Q., & Yu, F. (2021). Comparative efficacy of different exercise interventions on cognitive function in patients with MCI or dementia: A systematic review and network meta-analysis. *Journal of Sport and Health Science*. 10.1016/j.jshs.2021.05.003 PMC9068743 34004389

[CIT0021] Hunter, S. W., Divine, A., Madou, E., Omana, H., Hill, K. D., Johnson, A. M., Holmes, J. D., & Wittich, W. (2020). Executive function as a mediating factor between visual acuity and postural stability in cognitively healthy adults and adults with Alzheimer’s dementia. *Archives of Gerontology and Geriatrics*, *89*, 104078. 10.1016/j.archger.2020.104078 32388070

[CIT0022] Jacobs, J. M., Hammerman-Rozenberg, R., Maaravi, Y., Cohen, A., & Stessman, J. (2005). The impact of visual impairment on health, function and mortality. *Aging Clinical and Experimental Research*, *17*(4), 281–286. 16285193 10.1007/BF03324611

[CIT0023] Jutten, R. J., Peeters, C. F., Leijdesdorff, S. M., Visser, P. J., Maier, A. B., Terwee, C. B., Scheltens, P., & Sikkes, S. A. (2017). Detecting functional decline from normal aging to dementia: Development and validation of a short version of the Amsterdam IADL Questionnaire. *Alzheimer’s & Dementia: Diagnosis, Assessment & Disease Monitoring*, *8*(1), 26–35. 10.1016/j.dadm.2017.03.002 PMC5403784 28462387

[CIT0024] Karssemeijer, E. G. A (E.)., Aaronson, J. A (J.)., Bossers, W. J (W.)., Smits, T. (T.)., Olde Rikkert, M. G. M (M.)., & Kessels, R. P. C (R.). (2017). Positive effects of combined cognitive and physical exercise training on cognitive function in older adults with mild cognitive impairment or dementia: A meta-analysis. *Ageing Research Reviews*, *40*, 75–83. 10.1016/j.arr.2017.09.003 28912076

[CIT0025] Kearney, F. C., Harwood, R. H., Gladman, J. R., Lincoln, N., & Masud, T. (2013). The relationship between executive function and falls and gait abnormalities in older adults: A systematic review. *Dementia and Geriatric Cognitive Disorders*, *36*(1-2), 20–35. 23712088 10.1159/000350031

[CIT0026] Lam, F. M., Huang, M.-Z., Liao, L.-R., Chung, R. C., Kwok, T. C., & Pang, M. Y. (2018). Physical exercise improves strength, balance, mobility, and endurance in people with cognitive impairment and dementia: A systematic review. *Journal of Physiotherapy*, *64*(1), 4–15. 29289581 10.1016/j.jphys.2017.12.001

[CIT0027] Laver, K., Dyer, S., Whitehead, C., Clemson, L., & Crotty, M. (2016). Interventions to delay functional decline in people with dementia: A systematic review of systematic reviews. *BMJ Open*, *6*(4), e010767. 10.1136/bmjopen-2015-010767 PMC4854009 27121704

[CIT0028] Lawton, M. P., & Brody, E. M. (1969). Assessment of older people: Self-maintaining and instrumental activities of daily living. *The Gerontologist*, *9*(3 Part 1), 179–186. 10.1093/geront/9.3_Part_1.179 5349366

[CIT0029] Lee, H. S., Park, S. W., & Park, Y. J. (2016). Effects of physical activity programs on the improvement of dementia symptom: A meta-analysis. *BioMed Research International*, *2016*, 2920146. 10.1155/2016/2920146 27819000 PMC5081454

[CIT0030] Leng, M., Liang, B., Zhou, H., Zhang, P., Hu, M., Li, G., Li, F., & Chen, L. (2018). Effects of physical exercise on depressive symptoms in patients with cognitive impairment: A systematic review and meta-analysis. *The Journal of Nervous and Mental Disease*, *206*(10), 809–823. 30273278 10.1097/NMD.0000000000000887

[CIT0031] Lewis, K., Livsey, L., Naughton, R. J., & Burton, K. (2020). Exercise and dementia: What should we be recommending? *Quality in Ageing and Older Adults*, *21*(2), 109–127. 10.1108/QAOA-10-2019-0053

[CIT0032] Lewis, M., Peiris, C. L., & Shields, N. (2017). Long-term home and community-based exercise programs improve function in community-dwelling older people with cognitive impairment: A systematic review. *Journal of Physiotherapy*, *63*(1), 23–29. 10.1016/j.jphys.2016.11.005 27993488

[CIT0033] Li, X., Guo, R., Wei, Z., Jia, J., & Wei, C. (2019). Effectiveness of exercise programs on patients with dementia: A systematic review and meta-analysis of randomized controlled trials. *BioMed Research International*, *2019*, 1–16. 10.1155/2019/2308475 PMC6893254 31886182

[CIT0034] Lim, K. H.-L., Pysklywec, A., Plante, M., & Demers, L. (2019). The effectiveness of Tai Chi for short-term cognitive function improvement in the early stages of dementia in the elderly: A systematic literature review. *Clinical Interventions in Aging*, *14*, 827–839. 31190769 10.2147/CIA.S202055 PMC6512568

[CIT0035] Long, A., Robinson, K., Goldberg, S., & Gordon, A. L. (2019). Effectiveness of exercise interventions for adults over 65 with moderate-to-severe dementia in community settings: A systematic review. *European Geriatric Medicine*, *10*(6), 843–852. 34652766 10.1007/s41999-019-00236-7

[CIT0036] Lorenz, R. C., Matthias, K., Pieper, D., Wegewitz, U., Morche, J., Nocon, M., Rissling, O., Schirm, J., & Jacobs, A. (2019). A psychometric study found AMSTAR 2 to be a valid and moderately reliable appraisal tool. *Journal of Clinical Epidemiology*, *114*, 133–140. 31152864 10.1016/j.jclinepi.2019.05.028

[CIT0037] Machado, F. B., Silva, N., Farinatti, P., Poton, R., Ribeiro, Ó., & Carvalho, J. (2020). Effectiveness of multicomponent exercise interventions in older adults with dementia: A meta-analysis. *The Gerontologist*, 61(8), e449–e462. 10.1093/geront/gnaa091 PMC8599205 32652005

[CIT0038] Marques, C. L. S., Borgato, M. H., Moura Neto, E. d., Bazan, R., & Luvizutto, G. J. (2019). Physical therapy in patients with Alzheimer’s disease: A systematic review of randomized controlled clinical trials. *Fisioterapia e Pesquisa*, *26*(3), 311–321. 10.1590/1809-2950/18037226032019

[CIT0039] McDermott, O., Charlesworth, G., Hogervorst, E., Stoner, C., Moniz-Cook, E., Spector, A., Csipke, E., & Orrell, M. (2019). Psychosocial interventions for people with dementia: A synthesis of systematic reviews. *Aging & Mental Health*, *23*(4), 393–403. 29338323 10.1080/13607863.2017.1423031

[CIT0040] Meyer, C., & O’Keefe, F. (2020). Non-pharmacological interventions for people with dementia: A review of reviews. *Dementia (London, England)*, *19*(6), 1927–1954. 10.1177/1471301218813234 30526036

[CIT0041] Millán-Calenti, J. C., Tubío, J., Pita-Fernández, S., González-Abraldes, I., Lorenzo, T., Fernández-Arruty, T., & Maseda, A. (2010). Prevalence of functional disability in activities of daily living (ADL), instrumental activities of daily living (IADL) and associated factors, as predictors of morbidity and mortality. *Archives of Gerontology and Geriatrics*, *50*(3), 306–310. 10.1016/j.archger.2009.04.017 19520442

[CIT0042] Moher, D., Liberati, A., Tetzlaff, J., & Altman, D. G. (the PRISMA Group). PRISMA Group. (2009). Preferred reporting items for systematic reviews and meta-analyses: The PRISMA statement. *PLoS Medicine*, *6*(7), e1000097. 10.1371/journal.pmed.1000097 19621072 PMC2707599

[CIT0043] Nyman, S. R., Innes, A., & Heward, M. (2017). Social care and support needs of community-dwelling people with dementia and concurrent visual impairment. *Aging & Mental Health*, *21*(9), 961–967. 27215277 10.1080/13607863.2016.1186151

[CIT0044] Pieper, D., Antoine, S.-L., Mathes, T., Neugebauer, E. A., & Eikermann, M. (2014). Systematic review finds overlapping reviews were not mentioned in every other overview. *Journal of Clinical Epidemiology*, *67*(4), 368–375. 10.1016/j.jclinepi.2013.11.007 24581293

[CIT0045] Pitkälä, K., Savikko, N., Poysti, M., Strandberg, T., & Laakkonen, M.-L. (2013). Efficacy of physical exercise intervention on mobility and physical functioning in older people with dementia: A systematic review. *Experimental Gerontology*, *48*(1), 85–93. 22960590 10.1016/j.exger.2012.08.008

[CIT0046] Pollock, M., Fernandes, R. M., Becker, L. A., Pieper, D., & Hartling, L. (2018). Chapter V: Overviews of reviews. *Cochrane Handbook for Systematic Reviews of Interventions (version, 6)*.

[CIT0047] Ramzaoui, H., Faure, S., & Spotorno, S. (2018). Alzheimer’s disease, visual search, and instrumental activities of daily living: A review and a new perspective on attention and eye movements. *Journal of Alzheimer’s Disease*, *66*(3), 901–925. 10.3233/JAD-180043 30400086

[CIT0501] Rolland, Y., Pillard, F., Klapouszczak, A., Reynish, E., Thomas, D., Andrieu, S., Rivière, D., & Vellas, B. (2007). Exercise program for nursing home residents with Alzheimer’s disease: A 1‐year randomized, controlled trial. *Journal of the American Geriatrics Society*, *55*(2), 158–165. 17302650 10.1111/j.1532-5415.2007.01035.x

[CIT0048] Russ, J., Weyh, C., & Pilat, C. (2020). High-intensity exercise programs in people with dementia—A systematic review and meta-analysis. *German Journal of Exercise and Sport Research*, 51(1), 4–16. 1–13.

[CIT0049] Sultana, M., Bryant, D., Orange, J., Beedie, T., & Montero-Odasso, M. (2020). Effect of Wii Fit© exercise on balance of older adults with neurocognitive disorders: A meta-analysis. *Journal of Alzheimer’s Disease*, *75*(3), 817–826. 10.3233/JAD-191301 32310168

[CIT0050] Todd, S., Barr, S., Roberts, M., & Passmore, A. P. (2013). Survival in dementia and predictors of mortality: A review. *International Journal of Geriatric Psychiatry*, *28*(11), 1109–1124. 23526458 10.1002/gps.3946

[CIT0051] Toots, A., Littbrand, H., Lindelöf, N., Wiklund, R., Holmberg, H., Nordström, P., Lundin-Olsson, L., Gustafson, Y., & Rosendahl, E. (2016). Effects of a high-intensity functional exercise program on dependence in activities of daily living and balance in older adults with dementia. *Journal of the American Geriatrics Society*, *64*(1), 55–64. 26782852 10.1111/jgs.13880 PMC4722852

[CIT0052] Van Santen, J., Dröes, R.-M., Holstege, M., Henkemans, O. B., Van Rijn, A., De Vries, R., Van Straten, A., & Meiland, F. (2018). Effects of exergaming in people with dementia: Results of a systematic literature review. *Journal of Alzheimer’s Disease: JAD*, *63*(2), 741–760. 10.3233/JAD-170667 PMC5929299 29689716

[CIT0053] WHO. (2020). Dementia. https://www.who.int/news-room/fact-sheets/detail/dementia

[CIT0054] Wei, L., Chai, Q., Chen, J., Wang, Q., Bao, Y., Xu, W., & Ma, E. (2020). The impact of Tai Chi on cognitive rehabilitation of elder adults with mild cognitive impairment: A systematic review and meta-analysis. *Disability and Rehabilitation*, 1–10. 10.1080/09638288.2020.1830311 33043709

[CIT0055] Yang, J., Zhang, L., Tang, Q., Wang, F., Li, Y., Peng, H., & Wang, S. (2020). Tai Chi is effective in delaying cognitive decline in older adults with mild cognitive impairment: Evidence from a systematic review and meta-analysis. *Evidence-Based Complementary and Alternative Medicine: eCAM*, *2020*, 3620534. 10.1155/2020/3620534 32308706 PMC7132349

[CIT0056] Yeh, S.-W., Lin, L.-F., Chen, H.-C., Huang, L.-K., Hu, C.-J., Tam, K.-W., Kuan, Y.-C., & Hong, C.-H. (2021). High-intensity functional exercise in older adults with dementia: A systematic review and meta-analysis. *Clinical Rehabilitation*, *35*(2), 169–181. 33040592 10.1177/0269215520961637

[CIT0057] Zhang, Q., Hu, J., Wei, L., Cao, R., Ma, R., Song, H., & Jin, Y. (2019). Effects of traditional Chinese exercise on cognitive and psychological outcomes in older adults with mild cognitive impairment: A systematic review and meta-analysis. *Medicine*, *98*(7), e14581. 10.1097/MD.0000000000014581 30762810 PMC6408103

[CIT0058] Zhao, Y., Feng, H., Wu, X., Du, Y., Yang, X., Hu, M., Ning, H., Liao, L., Chen, H., & Zhao, Y. (2020). Effectiveness of exergaming in improving cognitive and physical function in people with mild cognitive impairment or dementia: Systematic review. *JMIR Serious Games*, *8*(2), e16841. 10.2196/16841 32602841 PMC7367532

[CIT0059] Zheng, G., Xia, R., Zhou, W., Tao, J., & Chen, L. (2016). Aerobic exercise ameliorates cognitive function in older adults with mild cognitive impairment: A systematic review and meta-analysis of randomised controlled trials. *British Journal of Sports Medicine*, *50*(23), 1443–1450. 10.1136/bjsports-2015-095699 27095745

[CIT0060] Zhou, X.-L., Wang, L.-N., Wang, J., Zhou, L., & Shen, X.-H. (2020). Effects of exercise interventions for specific cognitive domains in old adults with mild cognitive impairment: A meta-analysis and subgroup analysis of randomized controlled trials. *Medicine*, *99*(31), e20105. 10.1097/MD.0000000000020105 32756073 PMC7402775

[CIT0061] Zhu, L., Li, L., Wang, L., Jin, X., & Zhang, H. (2020). Physical activity for executive function and activities of daily living in AD patients: A systematic review and meta-analysis. *Frontiers in Psychology*, *11*, 3227. 10.3389/fpsyg.2020.560461 PMC7744293 33343442

[CIT0062] Zhu, X.-C., Yu, Y., Wang, H.-F., Jiang, T., Cao, L., Wang, C., Wang, J., Tan, C.-C., Meng, X.-F., Tan, L., & Yu, J.-T. (2015). Physiotherapy intervention in Alzheimer’s disease: Systematic review and meta-analysis. *Journal of Alzheimer’s Disease*, *44*(1), 163–174. 10.3233/JAD-141377 25201787

[CIT0063] Zou, L., Loprinzi, P. D., Yeung, A. S., Zeng, N., & Huang, T. (2019). The beneficial effects of mind-body exercises for people with mild cognitive impairment: A systematic review with meta-analysis. *Archives of Physical Medicine and Rehabilitation*, *100*(8), 1556–1573. 10.1016/j.apmr.2019.03.009 30986409

