# Supplementary material for: Does physical exercise improve the capacity for independent living in people with dementia or mild cognitive impairment: an overview of systematic reviews and meta-analyses
Source: Aging Ment Health. 2021 Dec 24;26(12):2317–27. doi: 10.1080/13607863.2021.2019192 (PMC9662184; doi:10.1080/13607863.2021.2019192)
Supplement: Supplemental Material [file CAMH_A_2019192_SM0729.docx]

1. **PRISMA Checklist**

| **Section/topic** | **#** | **Checklist item** | **Reported on page #** |
| --- | --- | --- | --- |
| **TITLE** | | |  |
| Title | 1 | Identify the report as a systematic review, meta-analysis, or both. | 1 |
| **ABSTRACT** | | |  |
| Structured summary | 2 | Provide a structured summary including, as applicable: background; objectives; data sources; study eligibility criteria, participants, and interventions; study appraisal and synthesis methods; results; limitations; conclusions and implications of key findings; systematic review registration number. | 1 |
| **INTRODUCTION** | | |  |
| Rationale | 3 | Describe the rationale for the review in the context of what is already known. | 1-2 |
| Objectives | 4 | Provide an explicit statement of questions being addressed with reference to participants, interventions, comparisons, outcomes, and study design (PICOS). | 2 |
| **METHODS** | | |  |
| Protocol and registration | 5 | Indicate if a review protocol exists, if and where it can be accessed (e.g., Web address), and, if available, provide registration information including registration number. | 2 |
| Eligibility criteria | 6 | Specify study characteristics (e.g., PICOS, length of follow-up) and report characteristics (e.g., years considered, language, publication status) used as criteria for eligibility, giving rationale. | 2 |
| Information sources | 7 | Describe all information sources (e.g., databases with dates of coverage, contact with study authors to identify additional studies) in the search and date last searched. | 2 |
| Search | 8 | Present full electronic search strategy for at least one database, including any limits used, such that it could be repeated. | 2 |
| Study selection | 9 | State the process for selecting studies (i.e., screening, eligibility, included in systematic review, and, if applicable, included in the meta-analysis). | 2 |
| Data collection process | 10 | Describe method of data extraction from reports (e.g., piloted forms, independently, in duplicate) and any processes for obtaining and confirming data from investigators. | 2 |
| Data items | 11 | List and define all variables for which data were sought (e.g., PICOS, funding sources) and any assumptions and simplifications made. | 2 |
| Risk of bias in individual studies | 12 | Describe methods used for assessing risk of bias of individual studies (including specification of whether this was done at the study or outcome level), and how this information is to be used in any data synthesis. | 2 |
| Summary measures | 13 | State the principal summary measures (e.g., risk ratio, difference in means). | 2 |
| Synthesis of results | 14 | Describe the methods of handling data and combining results of studies, if done, including measures of consistency (e.g., I^2^) for each meta-analysis. | 2 |

| Risk of bias across studies | 15 | Specify any assessment of risk of bias that may affect the cumulative evidence (e.g., publication bias, selective reporting within studies). | 2 |
| --- | --- | --- | --- |
| Additional analyses | 16 | Describe methods of additional analyses (e.g., sensitivity or subgroup analyses, meta-regression), if done, indicating which were pre-specified. | 2 |
| **RESULTS** | | |  |
| Study selection | 17 | Give numbers of studies screened, assessed for eligibility, and included in the review, with reasons for exclusions at each stage, ideally with a flow diagram. | 2-6 |
| Study characteristics | 18 | For each study, present characteristics for which data were extracted (e.g., study size, PICOS, follow-up period) and provide the citations. | 2-6 |
| Risk of bias within studies | 19 | Present data on risk of bias of each study and, if available, any outcome level assessment (see item 12). | 2-6 |
| Results of individual studies | 20 | For all outcomes considered (benefits or harms), present, for each study: (a) simple summary data for each intervention group (b) effect estimates and confidence intervals, ideally with a forest plot. | 2-6 |
| Synthesis of results | 21 | Present results of each meta-analysis done, including confidence intervals and measures of consistency. | 2-8 |
| Risk of bias across studies | 22 | Present results of any assessment of risk of bias across studies (see Item 15). | - |
| Additional analysis | 23 | Give results of additional analyses, if done (e.g., sensitivity or subgroup analyses, meta-regression [see Item 16]). | - |
| **DISCUSSION** | | |  |
| Summary of evidence | 24 | Summarize the main findings including the strength of evidence for each main outcome; consider their relevance to key groups (e.g., healthcare providers, users, and policy makers). | 8-9 |
| Limitations | 25 | Discuss limitations at study and outcome level (e.g., risk of bias), and at review-level (e.g., incomplete retrieval of identified research, reporting bias). | 9 |
| Conclusions | 26 | Provide a general interpretation of the results in the context of other evidence, and implications for future research. | 9 |
| **FUNDING** | | |  |
| Funding | 27 | Describe sources of funding for the systematic review and other support (e.g., supply of data); role of funders for the systematic review. | 9 |

| 1. **MEDLINE Search Strategy** 2. exp Dementia/ |
| --- |
| 1. exp Alzheimer Disease/ |
| 1. Alzheimer*.mp. |
| 1. exp mild cognitive impairment/ |
| 1. 1 or 2 or 3 or 4 |
| 1. exp Exercise/ or exp Exercise Therapy/ |
| 1. physical activity.mp. or Exercise/ |
| 1. cycling.mp. |
| 1. exp Exercise Therapy/ or gym*.mp. |
| 1. exp Physical Therapy Modalities/ |
| 1. Physical therapy.mp. |
| 1. 6 or 7 or 8 or 9 or 10 or 11 2. exp "Activities of Daily Living"/ |
| 1. ADL*.mp. |
| 1. Daily living activities.mp. |
| 1. Daily activities.mp. |
| 1. visual processing.mp. [mp=title, abstract, original title, name of substance word, subject heading word, floating sub-heading word, keyword heading word, organism supplementary concept word, protocol supplementary concept word, rare disease supplementary concept word, unique identifier, synonyms] |
| 1. (Eye Manifestations or "Visuospatial Ability" or "Visuospatial Function" or Visuospatial or "Visuospatial Attention" or "visual processing" or "Visuospatial processing").mp. [mp=title, abstract, original title, name of substance word, subject heading word, floating sub-heading word, keyword heading word, organism supplementary concept word, protocol supplementary concept word, rare disease supplementary concept word, unique identifier, synonyms] |
| 1. ((visual$ or vision or eye or eyes or eyesight or sight) adj5 (problem$ or disorder$ or impair$ or disabilit$ or loss or disease$ or defect$ or manifestation$ or screening or test$ or examination$)).mp. [mp=title, abstract, original title, name of substance word, subject heading word, floating sub-heading word, keyword heading word, organism supplementary concept word, protocol supplementary concept word, rare disease supplementary concept word, unique identifier, synonyms] |
| 1. exp Visual Acuity/ |
| 1. saccadic eye movements.mp. |
| 1. pupillary response.mp. |
| 1. exp Color Vision Defects/ or colour vision.mp. |
| 1. exp Visual Perception/ or exp Perceptual Masking/ or visual Masking.mp. |
| 1. exp Gait/ |
| 1. exp Walking/ |
| 1. walk*.mp. |
| 1. balance.mp. or exp Postural Balance/ 2. 13 or 14 or 15 or 16 or 17 or 18 or 19 or 20 or 21 or 22 or 23 or 24 or 25 or 26 or 27 or 28 3. 5 and 12 and 29 |

1. **Primary studies in each review**

**CCA for ADL: 4.8 %**

| **Reviews**      **Studies** | **Brett 2016** | **De Almeida**  **2020** | **Farhang 2019** | **Forbes 2015** | **Groot**  **2016** | **Karssemeijer**  **2017** | **Lam 2018** | **Lee 2016** | **Leng 2018** | **Lewis 2017** | **Li 2019** | **Machado 2020** | **Marques 2019** | **Russ 2020** | **Santen 2018** | **Wei 2020** | **Yeh 2021** | **Zhao 2020** | **Zhu 2015** | **Zhu**  **2020** |
| --- | --- | --- | --- | --- | --- | --- | --- | --- | --- | --- | --- | --- | --- | --- | --- | --- | --- | --- | --- | --- |
| Abd El-Kader and Al-Jiffri (2016) |  |  |  |  |  |  |  |  |  |  |  |  |  |  |  |  |  |  |  |  |
| Aguiar et al. (2014) |  |  |  |  |  |  |  |  |  |  |  |  |  |  |  |  |  |  |  |  |
| (Bamidis et al., 2015) |  |  |  |  |  |  |  |  |  |  |  |  |  |  |  |  |  |  |  |  |
| (Bürge et al., 2017) |  |  |  |  |  |  |  |  |  |  |  |  |  |  |  |  |  |  |  |  |
| (Callahan et al., 2017) |  |  |  |  |  |  |  |  |  |  |  |  |  |  |  |  |  |  |  |  |
| (Cancela et al., 2016) |  |  |  |  |  |  |  |  |  |  |  |  |  |  |  |  |  |  |  |  |
| (Chang, 2015) |  |  |  |  |  |  |  |  |  |  |  |  |  |  |  |  |  |  |  |  |
| (Conradsson et al., 2010) |  |  |  |  |  |  |  |  |  |  |  |  |  |  |  |  |  |  |  |  |
| (Cott et al., 2002) |  |  |  |  |  |  |  |  |  |  |  |  |  |  |  |  |  |  |  |  |
| (Dawson et al., 2019) |  |  |  |  |  |  |  |  |  |  |  |  |  |  |  |  |  |  |  |  |
| (de Souto Barreto et al., 2017) |  |  |  |  |  |  |  |  |  |  |  |  |  |  |  |  |  |  |  |  |
| (Eyre et al., 2017) |  |  |  |  |  |  |  |  |  |  |  |  |  |  |  |  |  |  |  |  |
| (Fogarty et al., 2016) |  |  |  |  |  |  |  |  |  |  |  |  |  |  |  |  |  |  |  |  |
| (Fonte et al., 2019) |  |  |  |  |  |  |  |  |  |  |  |  |  |  |  |  |  |  |  |  |
| (Francese et al., 1997) |  |  |  |  |  |  |  |  |  |  |  |  |  |  |  |  |  |  |  |  |
| (Graessel et al., 2011) |  |  |  |  |  |  |  |  |  |  |  |  |  |  |  |  |  |  |  |  |
| (Guan XH, 2016) |  |  |  |  |  |  |  |  |  |  |  |  |  |  |  |  |  |  |  |  |
| (Han et al., 2017) |  |  |  |  |  |  |  |  |  |  |  |  |  |  |  |  |  |  |  |  |
| (Hoffmann et al., 2016) |  |  |  |  |  |  |  |  |  |  |  |  |  |  |  |  |  |  |  |  |
| (Hokkanen et al., 2008) |  |  |  |  |  |  |  |  |  |  |  |  |  |  |  |  |  |  |  |  |
| (Holthoff et al., 2015) |  |  |  |  |  |  |  |  |  |  |  |  |  |  |  |  |  |  |  |  |
| (Karydaki et al., 2017) |  |  |  |  |  |  |  |  |  |  |  |  |  |  |  |  |  |  |  |  |
| (Kasai et al., 2010) |  |  |  |  |  |  |  |  |  |  |  |  |  |  |  |  |  |  |  |  |
| (Kwak et al., 2008) |  |  |  |  |  |  |  |  |  |  |  |  |  |  |  |  |  |  |  |  |
| (Lam et al., 2010) |  |  |  |  |  |  |  |  |  |  |  |  |  |  |  |  |  |  |  |  |
| (Lam et al., 2014) |  |  |  |  |  |  |  |  |  |  |  |  |  |  |  |  |  |  |  |  |
| (Lam et al., 2015) |  |  |  |  |  |  |  |  |  |  |  |  |  |  |  |  |  |  |  |  |
| (Lamb et al., 2018) |  |  |  |  |  |  |  |  |  |  |  |  |  |  |  |  |  |  |  |  |
| (Lin, 2017) |  |  |  |  |  |  |  |  |  |  |  |  |  |  |  |  |  |  |  |  |
| (Littbrand et al., 2009) |  |  |  |  |  |  |  |  |  |  |  |  |  |  |  |  |  |  |  |  |
| (Littbrand et al, 2011) |  |  |  |  |  |  |  |  |  |  |  |  |  |  |  |  |  |  |  |  |
| (Luttenberger et al., 2012) |  |  |  |  |  |  |  |  |  |  |  |  |  |  |  |  |  |  |  |  |
| (Maki et al., 2012) |  |  |  |  |  |  |  |  |  |  |  |  |  |  |  |  |  |  |  |  |
| (Maci et al., 2012) |  |  |  |  |  |  |  |  |  |  |  |  |  |  |  |  |  |  |  |  |
| (Nascimento et al., 2012) |  |  |  |  |  |  |  |  |  |  |  |  |  |  |  |  |  |  |  |  |
| (Padala et al., 2012) |  |  |  |  |  |  |  |  |  |  |  |  |  |  |  |  |  |  |  |  |
| (Padala et al., 2017) |  |  |  |  |  |  |  |  |  |  |  |  |  |  |  |  |  |  |  |  |
| (Pedroso et al.) |  |  |  |  |  |  |  |  |  |  |  |  |  |  |  |  |  |  |  |  |
| (Pitkälä et al., 2013) |  |  |  |  |  |  |  |  |  |  |  |  |  |  |  |  |  |  |  |  |
| (Roach et al., 2011) |  |  |  |  |  |  |  |  |  |  |  |  |  |  |  |  |  |  |  |  |
| (Rolland et al., 2007) |  |  |  |  |  |  |  |  |  |  |  |  |  |  |  |  |  |  |  |  |
| (Santana-Sosa et al., 2008) |  |  |  |  |  |  |  |  |  |  |  |  |  |  |  |  |  |  |  |  |
| (Singh et al., 2014) |  |  |  |  |  |  |  |  |  |  |  |  |  |  |  |  |  |  |  |  |
| (Siu and Lee, 2018) |  |  |  |  |  |  |  |  |  |  |  |  |  |  |  |  |  |  |  |  |
| (Steinberg et al., 2009) |  |  |  |  |  |  |  |  |  |  |  |  |  |  |  |  |  |  |  |  |
| (Stevens and Killeen, 2006) |  |  |  |  |  |  |  |  |  |  |  |  |  |  |  |  |  |  |  |  |
| (Sungkarat et al., 2017) |  |  |  |  |  |  |  |  |  |  |  |  |  |  |  |  |  |  |  |  |
| (Tappen, 1994) |  |  |  |  |  |  |  |  |  |  |  |  |  |  |  |  |  |  |  |  |
| (Telenius et al., 2015) |  |  |  |  |  |  |  |  |  |  |  |  |  |  |  |  |  |  |  |  |
| (Toots et al., 2016) |  |  |  |  |  |  |  |  |  |  |  |  |  |  |  |  |  |  |  |  |
| (Venturelli et al., 2011) |  |  |  |  |  |  |  |  |  |  |  |  |  |  |  |  |  |  |  |  |
| (Vidoni et al., 2019) |  |  |  |  |  |  |  |  |  |  |  |  |  |  |  |  |  |  |  |  |
| (Vreugdenhil et al., 2012) |  |  |  |  |  |  |  |  |  |  |  |  |  |  |  |  |  |  |  |  |
| (Wei and Ji, 2014) |  |  |  |  |  |  |  |  |  |  |  |  |  |  |  |  |  |  |  |  |
| (Wells et al., 2013) |  |  |  |  |  |  |  |  |  |  |  |  |  |  |  |  |  |  |  |  |
| (Wong et al., 2017) |  |  |  |  |  |  |  |  |  |  |  |  |  |  |  |  |  |  |  |  |
| (Yang et al., 2015) |  |  |  |  |  |  |  |  |  |  |  |  |  |  |  |  |  |  |  |  |
| (Yang et al., 2016) |  |  |  |  |  |  |  |  |  |  |  |  |  |  |  |  |  |  |  |  |
| (Yoon et al., 2013) |  |  |  |  |  |  |  |  |  |  |  |  |  |  |  |  |  |  |  |  |


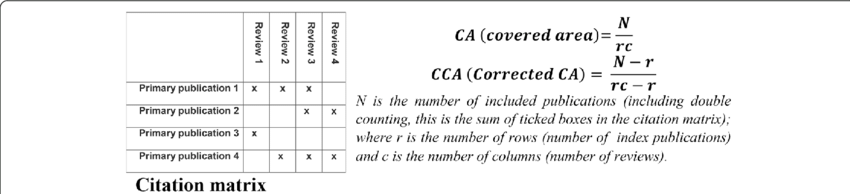


**CCA for Walking: 6.2%**

| **Reviews**      **Studies** | **Brett 2016** | **Farhang 2019** | **Lam 2018** | **Long 2019** | **Machado 2020** | **Russ 2020** | **Santen 2018** | **Yeh 2021** | **Zhu 2015** |
| --- | --- | --- | --- | --- | --- | --- | --- | --- | --- |
| (Aguiar et al., 2014) |  |  |  |  |  |  |  |  |  |
| (Arkin, 2003) |  |  |  |  |  |  |  |  |  |
| (Bossers et al., 2015) |  |  |  |  |  |  |  |  |  |
| (Cancela et al., 2016) |  |  |  |  |  |  |  |  |  |
| (Cott et al., 2002) |  |  |  |  |  |  |  |  |  |
| (Christofoletti et al., 2008) |  |  |  |  |  |  |  |  |  |
| (Coelho et al., 2013) |  |  |  |  |  |  |  |  |  |
| (de Souto Barreto et al., 2017) |  |  |  |  |  |  |  |  |  |
| (Fogarty et al., 2016) |  |  |  |  |  |  |  |  |  |
| (Garuffi et al., 2013) |  |  |  |  |  |  |  |  |  |
| (Hauer et al., 2012) |  |  |  |  |  |  |  |  |  |
| (Kemoun et al., 2010) |  |  |  |  |  |  |  |  |  |
| (Kovacs et al., 2013) |  |  |  |  |  |  |  |  |  |
| (Lü et al., 2016) |  |  |  |  |  |  |  |  |  |
| (Netz et al., 2007) |  |  |  |  |  |  |  |  |  |
| (Padala et al., 2012) |  |  |  |  |  |  |  |  |  |
| (Ries et al., 2010) |  |  |  |  |  |  |  |  |  |
| (Roach et al., 2011) |  |  |  |  |  |  |  |  |  |
| (Rolland et al., 2007) |  |  |  |  |  |  |  |  |  |
| (Suttanon et al., 2013) |  |  |  |  |  |  |  |  |  |
| (Schwenk et al., 2011) |  |  |  |  |  |  |  |  |  |
| (Toots et al., 2016) |  |  |  |  |  |  |  |  |  |
| (Telenius et al., 2015) |  |  |  |  |  |  |  |  |  |
| (Varela et al., 2012) |  |  |  |  |  |  |  |  |  |
| (Venturelli et al., 2011) |  |  |  |  |  |  |  |  |  |
| (Vreugdenhil et al., 2012) |  |  |  |  |  |  |  |  |  |

**CCA for Balance: 4.4 %**

| **Reviews**      **Studies** | **Brett 2016** | **Farhang 2019** | **Lam 2018** | **Lewis 2017** | **Machado 2020** | **Russ 2020** | **Santen 2018** | **Sultana 2020** | **Yeh 2021** | **Zhao 2020** | **Zhu 2015** |
| --- | --- | --- | --- | --- | --- | --- | --- | --- | --- | --- | --- |
| (Arcoverde et al., 2014) |  |  |  |  |  |  |  |  |  |  |  |
| (Bamidis et al., 2015) |  |  |  |  |  |  |  |  |  |  |  |
| (Ben-Sadoun et al., 2016) |  |  |  |  |  |  |  |  |  |  |  |
| (Canonici et al., 2012) |  |  |  |  |  |  |  |  |  |  |  |
| (Christofoletti et al., 2008) |  |  |  |  |  |  |  |  |  |  |  |
| (Chao et al., 2015) |  |  |  |  |  |  |  |  |  |  |  |
| (Fogarty et al., 2016) |  |  |  |  |  |  |  |  |  |  |  |
| (Hauer et al., 2012) |  |  |  |  |  |  |  |  |  |  |  |
| (Hernandez et al., 2010) |  |  |  |  |  |  |  |  |  |  |  |
| (Karydaki et al., 2017) |  |  |  |  |  |  |  |  |  |  |  |
| (Kwak et al., 2008) |  |  |  |  |  |  |  |  |  |  |  |
| (Lam et al., 2012) |  |  |  |  |  |  |  |  |  |  |  |
| (Lam et al., 2014) |  |  |  |  |  |  |  |  |  |  |  |
| (Lam, 2016) |  |  |  |  |  |  |  |  |  |  |  |
| (Lee, 2016) |  |  |  |  |  |  |  |  |  |  |  |
| (Littbrand et al., 2020) |  |  |  |  |  |  |  |  |  |  |  |
| (Padala et al., 2012) |  |  |  |  |  |  |  |  |  |  |  |
| (Padala et al., 2017) |  |  |  |  |  |  |  |  |  |  |  |
| (Ries et al., 2010) |  |  |  |  |  |  |  |  |  |  |  |
| (Rolland et al., 2007) |  |  |  |  |  |  |  |  |  |  |  |
| (Singh et al., 2013) |  |  |  |  |  |  |  |  |  |  |  |
| (Suttanon et al., 2013) |  |  |  |  |  |  |  |  |  |  |  |
| (Schwenk et al., 2011) |  |  |  |  |  |  |  |  |  |  |  |
| (Telenius et al., 2015) |  |  |  |  |  |  |  |  |  |  |  |
| (Toots et al., 2016) |  |  |  |  |  |  |  |  |  |  |  |
| (Vreugdenhil et al., 2012) |  |  |  |  |  |  |  |  |  |  |  |
| (Yoon et al., 2013) |  |  |  |  |  |  |  |  |  |  |  |

**CCA for Visuospatial: 6%**

| **Reviews**      **Studies** | **Cai 2020** | **Chan 2020** | **Farhang 2019** | **Lim 2019** | **Wei 2020** | **Yang 2020** | **Zhang 2019** | **Zhao 2020** | **Zheng 2016** | **Zhou 2020** | **Zou 2019** |
| --- | --- | --- | --- | --- | --- | --- | --- | --- | --- | --- | --- |
| (Aguiñaga, 2016) |  |  |  |  |  |  |  |  |  |  |  |
| (Barnes et al., 2013) |  |  |  |  |  |  |  |  |  |  |  |
| (Doi et al., 2017) |  |  |  |  |  |  |  |  |  |  |  |
| (Eyre et al., 2017) |  |  |  |  |  |  |  |  |  |  |  |
| (Lam et al., 2010) |  |  |  |  |  |  |  |  |  |  |  |
| (Lam et al., 2012) |  |  |  |  |  |  |  |  |  |  |  |
| (Lam et al., 2014) |  |  |  |  |  |  |  |  |  |  |  |
| (Liu et al., 2018) |  |  |  |  |  |  |  |  |  |  |  |
| (Mortimer et al., 2012) |  |  |  |  |  |  |  |  |  |  |  |
| (Mrakic-Sposta et al., 2018) |  |  |  |  |  |  |  |  |  |  |  |
| (Qi et al., 2019) |  |  |  |  |  |  |  |  |  |  |  |
| (Sungkarat et al., 2017) |  |  |  |  |  |  |  |  |  |  |  |
| (Sungkarat et al., 2018) |  |  |  |  |  |  |  |  |  |  |  |
| (Vreugdenhil et al., 2012) |  |  |  |  |  |  |  |  |  |  |  |
| (Yang et al., 2016) |  |  |  |  |  |  |  |  |  |  |  |
| (Zhu et al., 2018) |  |  |  |  |  |  |  |  |  |  |  |

References

ABD EL-KADER, S. M. & AL-JIFFRI, O. H. 2016. Aerobic exercise improves quality of life, psychological well-being and systemic inflammation in subjects with Alzheimer’s disease. *African health sciences,* 16**,** 1045-1055.

AGUIAR, P., MONTEIRO, L., FERES, A., GOMES, I. & MELO, A. 2014. Rivastigmine transdermal patch and physical exercises for Alzheimer's disease: a randomized clinical trial. *Current Alzheimer Research,* 11**,** 532-537.

AGUIÑAGA, S. 2016. *Latinos unique scenario, addressing cognitive impairment via dance.* University of Illinois at Chicago.

ARCOVERDE, C., DESLANDES, A., MORAES, H., ALMEIDA, C., ARAUJO, N. B. D., VASQUES, P. E., SILVEIRA, H. & LAKS, J. 2014. Treadmill training as an augmentation treatment for Alzheimer? s disease: a pilot randomized controlled study. *Arquivos de neuro-psiquiatria,* 72**,** 190-196.

ARKIN, S. M. 2003. Student-led exercise sessions yield significant fitness gains for Alzheimer's patients. *American Journal of Alzheimer's Disease & Other Dementias®,* 18**,** 159-170.

BAMIDIS, P. D., FISSLER, P., PAPAGEORGIOU, S. G., ZILIDOU, V., KONSTANTINIDIS, E. I., BILLIS, A. S., ROMANOPOULOU, E., KARAGIANNI, M., BERATIS, I. & TSAPANOU, A. 2015. Gains in cognition through combined cognitive and physical training: the role of training dosage and severity of neurocognitive disorder. *Frontiers in aging neuroscience,* 7**,** 152.

BARNES, D. E., SANTOS-MODESITT, W., POELKE, G., KRAMER, A. F., CASTRO, C., MIDDLETON, L. E. & YAFFE, K. 2013. The Mental Activity and eXercise (MAX) trial: a randomized controlled trial to enhance cognitive function in older adults. *JAMA internal medicine,* 173**,** 797-804.

BEN-SADOUN, G., SACCO, G., MANERA, V., BOURGEOIS, J., KÖNIG, A., FOULON, P., FOSTY, B., BREMOND, F., D’ARRIPE-LONGUEVILLE, F. & ROBERT, P. 2016. Physical and cognitive stimulation using an exergame in subjects with normal aging, mild and moderate cognitive impairment. *Journal of Alzheimer's Disease,* 53**,** 1299-1314.

BOSSERS, W. J., VAN DER WOUDE, L. H., BOERSMA, F., HORTOBÁGYI, T., SCHERDER, E. J. & VAN HEUVELEN, M. J. 2015. A 9-week aerobic and strength training program improves cognitive and motor function in patients with dementia: a randomized, controlled trial. *The American Journal of Geriatric Psychiatry,* 23**,** 1106-1116.

BÜRGE, E., BERCHTOLD, A., MAUPETIT, C., BOURQUIN, N. M.-P., VON GUNTEN, A., DUCRAUX, D., ZUMBACH, S., PEETERS, A. & KUHNE, N. 2017. Does physical exercise improve ADL capacities in people over 65 years with moderate or severe dementia hospitalized in an acute psychiatric setting? A multisite randomized clinical trial. *International psychogeriatrics,* 29**,** 323-332.

CALLAHAN, C. M., BOUSTANI, M. A., SCHMID, A. A., LAMANTIA, M. A., AUSTROM, M. G., MILLER, D. K., GAO, S., FERGUSON, D. Y., LANE, K. A. & HENDRIE, H. C. 2017. Targeting functional decline in Alzheimer disease: a randomized trial. *Annals of internal medicine,* 166**,** 164-171.

CANCELA, J. M., AYÁN, C., VARELA, S. & SEIJO, M. 2016. Effects of a long-term aerobic exercise intervention on institutionalized patients with dementia. *Journal of science and medicine in sport,* 19**,** 293-298.

CANONICI, A. P., ANDRADE, L. P. D., GOBBI, S., SANTOS‐GALDUROZ, R. F., GOBBI, L. T. B. & STELLA, F. 2012. Functional dependence and caregiver burden in Alzheimer's disease: a controlled trial on the benefits of motor intervention. *Psychogeriatrics,* 12**,** 186-192.

CHANG, C. H., WAN, W., ZH, Y., AND YAN, S. Y. 2015. Study on the intervention of aerobic training on alzheimer’s disease. . *Chin. J. Rehabil. Med. ,* 11**,** 1131–1134.

CHAO, Y.-Y., SCHERER, Y. K., MONTGOMERY, C. A., WU, Y.-W. & LUCKE, K. T. 2015. Physical and psychosocial effects of Wii Fit exergames use in assisted living residents: a pilot study. *Clinical nursing research,* 24**,** 589-603.

CHRISTOFOLETTI, G., OLIANI, M. M., GOBBI, S., STELLA, F., BUCKEN GOBBI, L. T. & RENATO CANINEU, P. 2008. A controlled clinical trial on the effects of motor intervention on balance and cognition in institutionalized elderly patients with dementia. *Clinical Rehabilitation,* 22**,** 618-626.

COELHO, F. G. D. M., ANDRADE, L. P., PEDROSO, R. V., SANTOS‐GALDUROZ, R. F., GOBBI, S., COSTA, J. L. R. & GOBBI, L. T. B. 2013. Multimodal exercise intervention improves frontal cognitive functions and gait in Alzheimer's disease: a controlled trial. *Geriatrics & gerontology international,* 13**,** 198-203.

CONRADSSON, M., LITTBRAND, H., LINDELÖF, N., GUSTAFSON, Y. & ROSENDAHL, E. 2010. Effects of a high-intensity functional exercise programme on depressive symptoms and psychological well-being among older people living in residential care facilities: a cluster-randomized controlled trial. *Aging & mental health,* 14**,** 565-576.

COTT, C. A., DAWSON, P., SIDANI, S. & WELLS, D. 2002. The effects of a walking/talking program on communication, ambulation, and functional status in residents with Alzheimer disease. *Alzheimer Disease & Associated Disorders,* 16**,** 81-87.

DAWSON, N., JUDGE, K. S. & GERHART, H. 2019. Improved functional performance in individuals with dementia after a moderate-intensity home-based exercise program: a randomized controlled trial. *Journal of Geriatric Physical Therapy,* 42**,** 18-27.

DE SOUTO BARRETO, P., CESARI, M., DENORMANDIE, P., ARMAINGAUD, D., VELLAS, B. & ROLLAND, Y. 2017. Exercise or social intervention for nursing home residents with dementia: a pilot randomized, controlled trial. *Journal of the american geriatrics society,* 65**,** E123-E129.

DOI, T., VERGHESE, J., MAKIZAKO, H., TSUTSUMIMOTO, K., HOTTA, R., NAKAKUBO, S., SUZUKI, T. & SHIMADA, H. 2017. Effects of cognitive leisure activity on cognition in mild cognitive impairment: results of a randomized controlled trial. *Journal of the American Medical Directors Association,* 18**,** 686-691.

EYRE, H. A., SIDDARTH, P., ACEVEDO, B., VAN DYK, K., PAHOLPAK, P., ERCOLI, L., CYR, N. S., YANG, H., KHALSA, D. S. & LAVRETSKY, H. 2017. A randomized controlled trial of Kundalini yoga in mild cognitive impairment. *International psychogeriatrics,* 29**,** 557-567.

FOGARTY, J. N., MURPHY, K. J., MCFARLANE, B., MONTERO-ODASSO, M., WELLS, J., TROYER, A. K., TRINH, D., GUTMANIS, I. & HANSEN, K. T. 2016. Taoist Tai Chi® and memory intervention for individuals with mild cognitive impairment. *Journal of aging and physical activity,* 24**,** 169-180.

FONTE, C., SMANIA, N., PEDRINOLLA, A., MUNARI, D., GANDOLFI, M., PICELLI, A., VARALTA, V., BENETTI, M. V., BRUGNERA, A. & FEDERICO, A. 2019. Comparison between physical and cognitive treatment in patients with MIC and Alzheimer’s disease. *Aging (Albany NY),* 11**,** 3138.

FRANCESE, T., SORRELL, J. & BUTLER, F. R. 1997. The effects of regular exercise on muscle strength and functional abilities of late stage Alzheimer's residents. *American Journal of Alzheimer's Disease,* 12**,** 122-127.

GARUFFI, M., COSTA, J. L. R., HERNÁNDEZ, S. S. S., VITAL, T. M., STEIN, A. M., SANTOS, J. G. D. & STELLA, F. 2013. Effects of resistance training on the performance of activities of daily living in patients with Alzheimer's disease. *Geriatrics & gerontology international,* 13**,** 322-328.

GRAESSEL, E., STEMMER, R., EICHENSEER, B., PICKEL, S., DONATH, C., KORNHUBER, J. & LUTTENBERGER, K. 2011. Non-pharmacological, multicomponent group therapy in patients with degenerative dementia: a 12-month randomized, controlled trial. *BMC medicine,* 9**,** 1-11.

GUAN XH, L. B. 2016. Effects of tai chi training on cognitive and social functions of Parkinson’s patients. *J Nurses Train,* 31(18):1684–1686.

HAN, J. W., LEE, H., HONG, J. W., KIM, K., KIM, T., BYUN, H. J., KO, J. W., YOUN, J. C., RYU, S.-H. & LEE, N.-J. 2017. Multimodal cognitive enhancement therapy for patients with mild cognitive impairment and mild dementia: a multi-center, randomized, controlled, double-blind, crossover trial. *Journal of Alzheimer's Disease,* 55**,** 787-796.

HAUER, K., SCHWENK, M., ZIESCHANG, T., ESSIG, M., BECKER, C. & OSTER, P. 2012. Physical training improves motor performance in people with dementia: a randomized controlled trial. *Journal of the American Geriatrics Society,* 60**,** 8-15.

HERNANDEZ, S. S., COELHO, F. G., GOBBI, S. & STELLA, F. 2010. Effects Of Physical Activity On Cognitive Functions, Balance And Risk Of Falls In Elderly Patients With Alzheimer's Dementia [efeitos De Um Programa De Atividade Física Nas Funções Cognitivas, Equilíbrio E Risco De Quedas Em Idosos Com Demência De Alzheimer]. *Revista brasileira de fisioterapia*.

HOFFMANN, K., SOBOL, N. A., FREDERIKSEN, K. S., BEYER, N., VOGEL, A., VESTERGAARD, K., BRÆNDGAARD, H., GOTTRUP, H., LOLK, A. & WERMUTH, L. 2016. Moderate-to-high intensity physical exercise in patients with Alzheimer’s disease: a randomized controlled trial. *Journal of Alzheimer's Disease,* 50**,** 443-453.

HOKKANEN, L., RANTALA, L., REMES, A. M., HÄRKÖNEN, B., VIRAMO, P. & WINBLAD, I. 2008. Dance and movement therapeutic methods in management of dementia: a randomized, controlled study. *Journal of the American Geriatrics Society,* 56**,** 771-772.

HOLTHOFF, V. A., MARSCHNER, K., SCHARF, M., STEDING, J., MEYER, S., KOCH, R. & DONIX, M. 2015. Effects of physical activity training in patients with Alzheimer’s dementia: results of a pilot RCT study. *PloS one,* 10**,** e0121478.

KARYDAKI, M., DIMAKOPOULOU, E., MARGIOTI, E., LYRAS, V., APOSTOLOPOULOS, X., PAPAGIANNI, M., VLACHOGIANNI, A., THEODORELOU, M. & SAKKA, P. 2017. Comparison of resistance and chair yoga training on subjective sleep quality in MCI women. *International Journal of Kinesiology and Sports Science,* 5**,** 26-34.

KASAI, J. Y. T., BUSSE, A. L., MAGALDI, R. M., SOCI, M. A., ROSA, P. D. M., CURIATI, J. A. E. & JACOB FILHO, W. 2010. Effects of Tai Chi Chuan on cognition of elderly women with mild cognitive impairment. *Einstein (São Paulo),* 8**,** 40-45.

KEMOUN, G., THIBAUD, M., ROUMAGNE, N., CARETTE, P., ALBINET, C., TOUSSAINT, L., PACCALIN, M. & DUGUÉ, B. 2010. Effects of a physical training programme on cognitive function and walking efficiency in elderly persons with dementia. *Dementia and geriatric cognitive disorders,* 29**,** 109-114.

KOVACS, E., SZTRUHAR JONASNE, I., KAROCZI, C., KORPOS, A. & GONDOS, T. 2013. Effects of a multimodal exercise program on balance, functional mobility and fall risk in older adults with cognitive impairment: a randomized controlled single-blind study. *Eur J Phys Rehabil Med,* 49**,** 639-648.

KWAK, Y.-S., UM, S.-Y., SON, T.-G. & KIM, D.-J. 2008. Effect of regular exercise on senile dementia patients. *International journal of sports medicine,* 29**,** 471-474.

LAM, L., CHAN, W., KWOK, T. & CHIU, H. 2014. Effectiveness of Tai Chi in maintenance of cognitive and functional abilities in mild cognitive impairment: a randomised controlled trial. *Hong Kong Med J,* 20**,** 20-23.

LAM, L. C., CHAU, R. C., WONG, B. M., FUNG, A. W., TAM, C. W., LEUNG, G. T., KWOK, T. C., LEUNG, T. Y., NG, S. P. & CHAN, W. M. 2012. A 1-year randomized controlled trial comparing mind body exercise (Tai Chi) with stretching and toning exercise on cognitive function in older Chinese adults at risk of cognitive decline. *Journal of the American Medical Directors Association,* 13**,** 568. e15-568. e20.

LAM, L. C., LUI, V. W., LUK, D. N., CHAU, R., SO, C., POON, V., TAM, P., CHING, R., LO, H. & CHIU, J. 2010. Effectiveness of an individualized functional training program on affective disturbances and functional skills in mild and moderate dementia—a randomized control trial. *International Journal of Geriatric Psychiatry: A journal of the psychiatry of late life and allied sciences,* 25**,** 133-141.

LAM, L. C.-W., CHAN, W. C., LEUNG, T., FUNG, A. W.-T. & LEUNG, E. M.-F. 2015. Would older adults with mild cognitive impairment adhere to and benefit from a structured lifestyle activity intervention to enhance cognition?: a cluster randomized controlled trial. *PloS one,* 10**,** e0118173.

LAM, M. H. 2016. The effect of whole body vibration on physical functioning in older adults.

LAMB, S. E., SHEEHAN, B., ATHERTON, N., NICHOLS, V., COLLINS, H., MISTRY, D., DOSANJH, S., SLOWTHER, A. M., KHAN, I. & PETROU, S. 2018. Dementia And Physical Activity (DAPA) trial of moderate to high intensity exercise training for people with dementia: randomised controlled trial. *bmj,* 361.

LEE, G.-H. 2016. Effects of virtual reality exercise program on balance, emotion and quality of life in patients with cognitive decline. *The Journal of Korean Physical Therapy,* 28**,** 355-363.

LIN, Q. 2017. Effect of Ba Duan Jin fitness exercise on elderly patients with mild cog- nitive impairment. . *Chinese J Gerontol,* 37**,** 3557–3560

.

LITTBRAND, H., LUNDIN‐OLSSON, L., GUSTAFSON, Y. & ROSENDAHL, E. 2009. The effect of a high‐intensity functional exercise program on activities of daily living: a randomized controlled trial in residential care facilities. *Journal of the American Geriatrics Society,* 57**,** 1741-1749.

LIU, T., GUO, S. & BAI, S. 2018. Effect of Baduanjin on cognition in patients with mild cognitive impairment. *Chinese J Rehabil Theory Practice,* 24**,** 854-9.

LÜ, J., SUN, M., LIANG, L., FENG, Y., PAN, X. & LIU, Y. 2016. Effects of momentum-based dumbbell training on cognitive function in older adults with mild cognitive impairment: a pilot randomized controlled trial. *Clinical interventions in aging,* 11**,** 9.

LUTTENBERGER, K., DONATH, C., UTER, W. & GRAESSEL, E. 2012. Effects of multimodal nondrug therapy on dementia symptoms and need for care in nursing home residents with degenerative dementia: a randomized‐controlled study with 6‐month follow‐up. *Journal of the American Geriatrics Society,* 60**,** 830-840.

MACI, T., PIRA, F. L., QUATTROCCHI, G., NUOVO, S. D., PERCIAVALLE, V. & ZAPPIA, M. 2012. Physical and cognitive stimulation in Alzheimer Disease. the GAIA Project: a pilot study. *American Journal of Alzheimer's Disease & Other Dementias®,* 27**,** 107-113.

MAKI, Y., URA, C., YAMAGUCHI, T., MURAI, T., ISAHAI, M., KAIHO, A., YAMAGAMI, T., TANAKA, S., MIYAMAE, F. & SUGIYAMA, M. 2012. Effects of intervention using a community‐based walking program for prevention of mental decline: A randomized controlled trial. *Journal of the American Geriatrics Society,* 60**,** 505-510.

MORTIMER, J. A., DING, D., BORENSTEIN, A. R., DECARLI, C., GUO, Q., WU, Y., ZHAO, Q. & CHU, S. 2012. Changes in brain volume and cognition in a randomized trial of exercise and social interaction in a community-based sample of non-demented Chinese elders. *Journal of Alzheimer's Disease,* 30**,** 757-766.

MRAKIC-SPOSTA, S., DI SANTO, S. G., FRANCHINI, F., ARLATI, S., ZANGIACOMI, A., GRECI, L., MORETTI, S., JESUTHASAN, N., MARZORATI, M. & RIZZO, G. 2018. Effects of combined physical and cognitive virtual reality-based training on cognitive impairment and oxidative stress in MCI patients: a pilot study. *Frontiers in aging neuroscience,* 10**,** 282.

NASCIMENTO, C., TEIXEIRA, C. V., GOBBI, L. T., GOBBI, S. & STELLA, F. 2012. A controlled clinical trial on the effects of exercise on neuropsychiatric disorders and instrumental activities in women with Alzheimer's disease. *Brazilian Journal of Physical Therapy,* 16**,** 197-204.

NETZ, Y., AXELRAD, S. & ARGOV, E. 2007. Group physical activity for demented older adults—feasibility and effectiveness. *Clinical rehabilitation,* 21**,** 977-986.

PADALA, K. P., PADALA, P. R., LENSING, S. Y., DENNIS, R. A., BOPP, M. M., ROBERSON, P. K. & SULLIVAN, D. H. 2017. Home-based exercise program improves balance and fear of falling in community-dwelling older adults with mild Alzheimer’s disease: a pilot study. *Journal of Alzheimer's disease,* 59**,** 565-574.

PADALA, K. P., PADALA, P. R., MALLOY, T. R., GESKE, J. A., DUBBERT, P. M., DENNIS, R. A., GARNER, K. K., BOPP, M. M., BURKE, W. J. & SULLIVAN, D. H. 2012. Wii-fit for improving gait and balance in an assisted living facility: a pilot study. *Journal of aging research,* 2012.

PEDROSO, R. V., AYÁN, C., FRAGA, F. J., SILVA, J. M. C. & SANTOS-GALDURÒZ, R. F. Effects of Functional-Task Training on Older Adults With Alzheimer’s. *Journal of Aging and Physical Activity*.

PITKÄLÄ, K. H., PÖYSTI, M. M., LAAKKONEN, M.-L., TILVIS, R. S., SAVIKKO, N., KAUTIAINEN, H. & STRANDBERG, T. E. 2013. Effects of the Finnish Alzheimer disease exercise trial (FINALEX): a randomized controlled trial. *JAMA internal medicine,* 173**,** 894-901.

QI, M., ZHU, Y., ZHANG, L., WU, T. & WANG, J. 2019. The effect of aerobic dance intervention on brain spontaneous activity in older adults with mild cognitive impairment: A resting‑state functional MRI study. *Experimental and therapeutic medicine,* 17**,** 715-722.

RIES, J. D., DRAKE, J. M. & MARINO, C. 2010. A small-group functional balance intervention for individuals with Alzheimer disease: a pilot study. *Journal of Neurologic Physical Therapy,* 34**,** 3-10.

ROACH, K. E., TAPPEN, R. M., KIRK-SANCHEZ, N., WILLIAMS, C. L. & LOEWENSTEIN, D. 2011. A randomized controlled trial of an activity specific exercise program for individuals with Alzheimer disease in long-term care settings. *Journal of geriatric physical therapy (2001),* 34**,** 50.

ROLLAND, Y., PILLARD, F., KLAPOUSZCZAK, A., REYNISH, E., THOMAS, D., ANDRIEU, S., RIVIÈRE, D. & VELLAS, B. 2007. Exercise program for nursing home residents with Alzheimer's disease: A 1‐year randomized, controlled trial. *Journal of the American Geriatrics Society,* 55**,** 158-165.

SANTANA-SOSA, E., BARRIOPEDRO, M., LOPEZ-MOJARES, L. M., PÉREZ, M. & LUCIA, A. 2008. Exercise training is beneficial for Alzheimer's patients. *International journal of sports medicine,* 29**,** 845.

SCHWENK, M., SCHMIDT, M., PFISTERER, M., OSTER, P. & HAUER, K. 2011. Rollator use adversely impacts on assessment of gait and mobility during geriatric rehabilitation. *Journal of rehabilitation medicine,* 43**,** 424-429.

SINGH, D. K. A., NORDIN, N. A. M., ABD AZIZ, N. A., LIM, B. K. & SOH, L. C. 2013. Effects of substituting a portion of standard physiotherapy time with virtual reality games among community-dwelling stroke survivors. *BMC neurology,* 13**,** 1-7.

SINGH, M. A. F., GATES, N., SAIGAL, N., WILSON, G. C., MEIKLEJOHN, J., BRODATY, H., WEN, W., SINGH, N., BAUNE, B. T. & SUO, C. 2014. The Study of Mental and Resistance Training (SMART) study—resistance training and/or cognitive training in mild cognitive impairment: a randomized, double-blind, double-sham controlled trial. *Journal of the American Medical Directors Association,* 15**,** 873-880.

SIU, M.-Y. & LEE, D. T. 2018. Effects of tai chi on cognition and instrumental activities of daily living in community dwelling older people with mild cognitive impairment. *BMC geriatrics,* 18**,** 1-10.

STEINBERG, M., LEOUTSAKOS, J. M. S., PODEWILS, L. J. & LYKETSOS, C. 2009. Evaluation of a home‐based exercise program in the treatment of Alzheimer's disease: The Maximizing Independence in Dementia (MIND) study. *International Journal of Geriatric Psychiatry: A journal of the psychiatry of late life and allied sciences,* 24**,** 680-685.

STEVENS, J. & KILLEEN, M. 2006. A randomised controlled trial testing the impact of exercise on cognitive symptoms and disability of residents with dementia. *Contemporary nurse,* 21**,** 32-40.

SUNGKARAT, S., BORIPUNTAKUL, S., CHATTIPAKORN, N., WATCHARASAKSILP, K. & LORD, S. R. 2017. Effects of Tai Chi on cognition and fall risk in older adults with mild cognitive impairment: a randomized controlled trial. *Journal of the American Geriatrics Society,* 65**,** 721-727.

SUNGKARAT, S., BORIPUNTAKUL, S., KUMFU, S., LORD, S. R. & CHATTIPAKORN, N. 2018. Tai Chi improves cognition and plasma BDNF in older adults with mild cognitive impairment: a randomized controlled trial. *Neurorehabilitation and neural repair,* 32**,** 142-149.

SUTTANON, P., HILL, K. D., SAID, C. M., WILLIAMS, S. B., BYRNE, K. N., LOGIUDICE, D., LAUTENSCHLAGER, N. T. & DODD, K. J. 2013. Feasibility, safety and preliminary evidence of the effectiveness of a home-based exercise programme for older people with Alzheimer’s disease: a pilot randomized controlled trial. *Clinical rehabilitation,* 27**,** 427-438.

TAPPEN, R. M. 1994. The effect of skill training on functional abilities of nursing home residents with dementia. *Research in nursing & health,* 17**,** 159-165.

TELENIUS, E. W., ENGEDAL, K. & BERGLAND, A. 2015. Effect of a high-intensity exercise program on physical function and mental health in nursing home residents with dementia: an assessor blinded randomized controlled trial. *PloS one,* 10**,** e0126102.

TOOTS, A., LITTBRAND, H., LINDELÖF, N., WIKLUND, R., HOLMBERG, H., NORDSTRÖM, P., LUNDIN‐OLSSON, L., GUSTAFSON, Y. & ROSENDAHL, E. 2016. Effects of a high‐intensity functional exercise program on dependence in activities of daily living and balance in older adults with dementia. *Journal of the American Geriatrics Society,* 64**,** 55-64.

VARELA, S., AYÁN, C., CANCELA, J. M. & MARTÍN, V. 2012. Effects of two different intensities of aerobic exercise on elderly people with mild cognitive impairment: a randomized pilot study. *Clinical rehabilitation,* 26**,** 442-450.

VENTURELLI, M., SCARSINI, R. & SCHENA, F. 2011. Six-month walking program changes cognitive and ADL performance in patients with Alzheimer. *American Journal of Alzheimer's Disease & Other Dementias®,* 26**,** 381-388.

VIDONI, E. D., PERALES, J., ALSHEHRI, M., GILES, A.-M., SIENGSUKON, C. F. & BURNS, J. M. 2019. Aerobic exercise sustains performance of instrumental activities of daily living in early-stage Alzheimer’s disease. *Journal of geriatric physical therapy (2001),* 42**,** E129.

VREUGDENHIL, A., CANNELL, J., DAVIES, A. & RAZAY, G. 2012. A community‐based exercise programme to improve functional ability in people with Alzheimer’s disease: A randomized controlled trial. *Scandinavian journal of caring sciences,* 26**,** 12-19.

WEI, X.-H. & JI, L.-L. 2014. Effect of handball training on cognitive ability in elderly with mild cognitive impairment. *Neuroscience letters,* 566**,** 98-101.

WELLS, R. E., YEH, G. Y., KERR, C. E., WOLKIN, J., DAVIS, R. B., TAN, Y., SPAETH, R., WALL, R. B., WALSH, J. & KAPTCHUK, T. J. 2013. Meditation's impact on default mode network and hippocampus in mild cognitive impairment: a pilot study. *Neuroscience letters,* 556**,** 15-19.

WONG, W. P., COLES, J., CHAMBERS, R., WU, D. B.-C. & HASSED, C. 2017. The effects of mindfulness on older adults with mild cognitive impairment. *Journal of Alzheimer's disease reports,* 1**,** 181-193.

YANG, H., LEAVER, A. M., SIDDARTH, P., PAHOLPAK, P., ERCOLI, L., ST CYR, N. M., EYRE, H. A., NARR, K. L., KHALSA, D. S. & LAVRETSKY, H. 2016. Neurochemical and neuroanatomical plasticity following memory training and yoga interventions in older adults with mild cognitive impairment. *Frontiers in aging neuroscience,* 8**,** 277.

YANG, S.-Y., SHAN, C.-L., QING, H., WANG, W., ZHU, Y., YIN, M.-M., MACHADO, S., YUAN, T.-F. & WU, T. 2015. The effects of aerobic exercise on cognitive function of Alzheimer’s disease patients. *CNS & Neurological Disorders-Drug Targets (Formerly Current Drug Targets-CNS & Neurological Disorders),* 14**,** 1292-1297.

YOON, J. E., LEE, S. M., LIM, H. S., KIM, T. H., JEON, J. K. & MUN, M. H. 2013. The effects of cognitive activity combined with active extremity exercise on balance, walking activity, memory level and quality of life of an older adult sample with dementia. *Journal of physical therapy science,* 25**,** 1601-1604.

ZHU, Y., WU, H., QI, M., WANG, S., ZHANG, Q., ZHOU, L., WANG, S., WANG, W., WU, T. & XIAO, M. 2018. Effects of a specially designed aerobic dance routine on mild cognitive impairment. *Clinical interventions in aging,* 13**,** 1691.

1. **Characteristics of reviews**

| **Review** | **Number of studies** | **Participants** | **Interventions**  **(length x frequency x duration)** | **Outcome measures** | **Main findings** |
| --- | --- | --- | --- | --- | --- |
| 1. Almeida, 2020 | n=16 | Patients with dementia (n=1129)  Mean age: 77.3 (from 51 to 99)  Gender: 49% female | Experimental: Physical activities, strength, endurance and flexibility exercises (20 min-12 hr x 1-7 days/wk x 8-104 wk)  Control: Usual care, social visits, conversation only, no intervention, education | ADL: Katz Index, ADCS-ADL, BI, The 16-item self-reported assessment tool,  LBS, IDDAD  Balance: FRT | Effect sizes (95% Confidence Interval)  SMD = ADL: 0.80 (0.53, 1.07), Balance: 2.24 (1.80, 2.68)  No of studies in ES: ADL: 2 (n=210), Balance: 2 (n=80)  I^2^ (%) = ADL: 99, Balance: 97  P=ADL: 0.00, Balance: 0.00   - Data from six studies (n=325) examined the effect of home-based exercise on ADL skills. Only one study found that doing exercise at home improved the performance of daily activities (P=0.04), whilst the other five studies reported a non-significant difference (P > 0.05). Only three studies reported minor adverse events related to home-based exercises (for example, stiffness, dizziness, headache). - Overall, the results demonstrated significant improvements in balance and mobility. |
| 1. Brett, 2016 | n=12 | Patients with dementia (n=901)  Mean age: 82.6  Gender: NR | Experimental: Multimodal exercises, walking, dance and hand exercises (30-120 min x 2-7 days/wk x 4-52 wk)  Control: Social visits, group reading, usual care, no intervention | ADL: Katz Index of ADLs, BI, ACIF  Walking: 6MWT, 2MWT, Locometer  Balance: BBS, One-leg balance test | No meta-analysis The effect of exercise on ADLs was investigated in five studies (n=406). Although statistically more improvement was observed in the experimental group than in the control group (P<.05) in three of these studies, non-significant results were found in one study (P >.05). Interestingly, another study reported that both groups had a worsening result in their performance of ADLs, but this was more in the control group.   - Walking ability was also used as the outcome measure in five studies (n=318). Four of the studies reported that exercise significantly improved the walking function, whereas the results of another study showed a non-significant impact of exercise on walking. - Balance was assessed in two studies (n=151). Although BBS improved significantly in both groups in one study (P <.05), another study did not find a significant change in either group (P >.05). |
| 1. Cai, 2020 | n=19 | Patients with dementia and MCI (n=1970)  Mean age: 66 to 82  Gender: 68% female | Experimental: tai chi (20-120 min x 1-5 days/wk x 10-52 wk)  Control: Health education, stretching exercise, social activities, and no intervention | Visuospatial function:  CDT, BDT | Effect sizes (95% Confidence Interval)  SMD = 0.03 (−0.28, 0.33)  No of studies in ES: 3 (n=200)  I^2^ (%) = 55  P=0.7   - Results showed that tai chi exercise had no significant effect on visuospatial ability. |
| 1. Chan, 2020 | n=5 | Patients with MCI (n=358)  Mean age: from 67 to 76  Gender: 67% female | Experimental: Dance interventions (25-60 min x 1-3 days/wk x 12-40 wk)  Control: Education, no intervention | Visuospatial function:  TMT | Effect sizes (95% Confidence Interval)  SMD = 0.16 (0.01, 0.32)  No of studies in ES: 4 (n=229)  I^2^ (%) = 0  P=0.03   - According to the meta-analysis results based on data from four studies, a significant improvement was found in the visuospatial function. |
| 1. Farhang, 2019 | n=9 | Patients with MCI (n=710) Mean age: 65 to 75 Gender: NR | Experimental: Mind-body interventions, yoga and tai chi (30-90 min x 1-7 days/wk x 6-26 wk)  Control: Education, no intervention, relaxation, stretching, usual care, memory enhancement training | ADL: B-ADL-25  Walking: Speed  Balance: BBS  Visuospatial function: BDT, ANT | No meta-analysis   - In a study in which ADL was used as the outcome measure (n=14), it was reported that mild body exercises significantly improved ADL performance. - Walking was evaluated in only one study, and it was found that tai chi did not positively affect walking (n=48). - Although one study (n=49) found positive results for balance, two studies (n=437) reported that exercise did not improve balance significantly. - Three studies (n=170) reported positive effects of yoga and tai chi on visuospatial ability. |
| 1. Forbes, 2015 | n=16 | Patients with dementia (n=937)  Mean age: 74.3  Gender:  69% female | Experimental: Cycling, walking, strength, aerobic, non-aerobic and combined physical activities (20-75 min x 1-5 days/wk x 2-78 wk)  Control: Usual care, social activities, no intervention | ADL: Katz Index of ADLs, BI, CADS | Effect sizes (95% Confidence Interval)  SMD = 0.68 (0.08, 1.27)  No of studies in ES: 6 (n=289)  I^2^ (%) = 77  P=0.03   - In six studies, the effect of exercise on ADLs was investigated. The performance of ADLs in the exercise group was improved more than usual care. No adverse event was reported during exercise. It was suggested that doing exercise could increase independence in performing daily activities. |
| 1. Groot, 2016 | n=18 | Patients with dementia (n=802)  Mean age: 79.7 ± 4.2 Gender: 68% female | Experimental: Aerobic, non-aerobic and combined physical activities (40-840 min/wk x 6-52 wk)  Control: Usual care, social visits, home safety training | ADL: JTT, BI, IADL | Effect sizes (95% Confidence Interval)  SMD = 1.18 (0.57, 1.79)  No of studies in ES: 4 (n=118)  I^2^ (%) = not reported  P<0.01   - A positive overall random effect of physical activity on ADL was found in the meta-analysis of four studies. |
| 1. Karssemeijer, 2017 | n=10 | Patients with dementia, AD and MCI (n=742)  Mean age: 72.1  Gender: 59% female | Experimental: Combined cognitive and physical exercises (30-120 min x 2-6 days/wk x 8-52 wk)  Control: Usual care, social visits, home safety training | ADL: DAD-ADL, E-ADL, ADCS-ADL, Bayer ADL | Effect sizes (95% Confidence Interval)  SMD = 0.65 (0.09, 1.21)  No of studies in ES: 4 (n=302)  I^2^ (%) = 80  P<0.01   - Meta-analysis results, including the results of four studies, showed a moderate to large positive effect of physical exercise interventions on ADLs. After removal of two studies, the effect size was still moderate-to-large but without the heterogeneity (SMD: 0.75, I^2^= 0%, P<0.01). |
| 1. Lam, 2018 | n=43 | Patients with dementia and MCI (n=3988)  Mean age: 68 to 89  Gender: NR | Experimental: Aerobic exercise, walking, dual-task walking, multimodal, stretching (30-150 min x 1-7 days/wk x 12-52 wk)  Control: NR | ADL: BI, Performance test of ADL, Katz ADL score, JTT  Walking: 2MWT, 6MWT, step length, speed  Balance BBS, TB, One-leg balance, FRT | Effect sizes (95% Confidence Interval)  MD = ADL: 9.59 (3.02, 16.16), Balance BBS: 3.61 (0.26, 6.95), Balance FRT: 3.85 (2.18, 5.53), 6MWT: 49.54 (17.70, 81.38), Gait speed: 0.13 (0.03, 0.24)  n (ES) = ADL: 4, Balance BBS: 6, Balance FRT: 4, 6MWT: 7, Gait speed: 7  I^2^ (%) = ADL: 89, Balance BBS: 91, Balance FRT: 0, 6MWT: 79, Gait speed: 90  P= ADL: 0.004, Balance BBS: 0.03, Balance FRT: 0.49, 6MWT: 0.002, Gait speed: 0.01   - Data from 22 studies were associated with the effect of exercise on ADL and 16 of them reported a significant positive effect of exercise. Meta-analysis of four studies (n=237) found that exercise significantly improved ADL performance. - The meta-analysis results also showed the positive effects of exercise on walking speed (n=568), walking distance (n=402) and balance (n=722). |
| 1. Lee, 2016 | n=9 | Patients with dementia (n=228)  Mean age: NR  Gender: NR | Experimental: Dance therapy, tai chi, strength and balance exercises, walking (30-150 min x 1-7 days/wk x 12-52 wk)  Control: NR | ADL: BI, ADL, IADL | Effect sizes (95% Confidence Interval)  SMD = 0.73 (0.23, 1.23)  n (ES) = 5  I^2^ (%) = NR  P=0.004   - In four studies, the effect of physical activity on ADL was investigated. The review found that physical activity was statistically significantly effective in improving the performance of ADLs. |
| 1. Leng, 2018 | n=21 | Patients with dementia, AD and MCI (n=2589)  Mean age: 67 to 89  Gender: 64% female | Experimental: Resistance training, yoga, tai chi, cycling, walking, strength, balance and aerobic exercises (15-90 min x 1-7 days/wk x 6-64 wk)  Control: Usual care, stretching exercise, social and cognitive activities | ADL: BI, Katz Index of ADLs, ADCS-ADL, CDAD-IADL | Effect sizes (95% Confidence Interval)  SMD = 0.27 (0.12, 0.43)  No of studies in ES: 4 (n=652)  I^2^ (%) = 0  P= 0.0005   - Data regarding the effect of exercise on ADL were available in five studies. All five studies reported the positive effects of exercise. Meta-analysis of four studies showed that exercise significantly improved ADL. Another study also reported a significant improvement in ADL by decreasing the score (n = 94; SMD = −1.32; 95% CI, −1.77 to −0.87; p < 0.00001). |
| 1. Lewis, 2017 | n=7 | Patients with dementia (n=945)  Mean age: 74 to 82  Gender: 64% female | Experimental: Long-term multicomponent exercise, treadmill walking, chair-based exercise (15-90 min x 1-7 days/wk x 16-52 wk)  Control: Usual care, social activity, education | ADL: ADL, IADL, FIM  Balance: SPPB, BBS, FRT | Effect sizes (95% Confidence Interval)  SMD = ADL: 0.77 (0.17, 1.37), IADL: 0.44 (0.03, 0.86),  MD= Balance: 5.2 (0.5, 9.9)  n (ES) = ADL: 3, IADL: 2, Balance: 3  I^2^ (%) = ADL: 67, IADL: 42, Balance: 76  P= ADL: 0.01, IADL: 0.04, Balance: 0.03   - Meta-analysis results on three studies (n=180), showed that long-term multicomponent exercises significantly improved ADL. Similarly, data from two studies (n=225) in which IADLs were assessed found statistically significant improvements in the exercise group. - The review also reported that multicomponent exercises had a significant effect on balance (n=48, P=0.03). |
| 1. Li, 2019 | n=20 | Patients with dementia (n=2051)  Mean age:  70.5 to 87.9  Gender: 60% female | Experimental: Strength and balance exercises including walking, squats, and trunk exercises, walking (6-52 wks)  Time and frequency: Not reported.  Control: Usual care, music therapy, education | ADL: Bristol ADL, ADCS-ADL, BI, FIM, IADL, CADS | Effect sizes (95% Confidence Interval)  SMD = 0.50 (-0.03, 1.02)  No of studies in ES: 11 (n=1502)  I^2^ (%) = 95  P=0.066   - According to the meta-analysis results, it was reported that exercise had no significant effect on the performance of ADLs. However, a significant result was found favouring exercise by excluding a study in the subgroup analysis performed to find the source of high heterogeneity (SMD: 0.87; 95% CI: 0.19 to 1.54; P = 0.012). |
| 1. Lim, 2019 | n=9 | Patients with dementia (n=656)  Mean age: 78  Gender: NR | Experimental: tai chi chuan (20-60 min x 1-4 days/wk x 8-52 wk)  Control: Education, physical activity, craft-based exercises | Visuospatial function:  BDT | No meta-analysis   - The effect of exercise on visuospatial function was investigated only in a high-quality study (n=66). It was found that the tai chi exercise was significantly more effective compared with the control group (P=0.01). |
| 1. Long, 2019 | n=8 | Patients with moderate-to-severe dementia (n=819)  Mean age: ≥65  Gender: NR | Experimental: Cycling, multicomponent exercises, walking (15-60 min x 2-7 days/wk x 15-65 wk)  Control: Usual care, social conversation | Walking: 2MWT, 6MWT, 10MWT, TUG | No meta-analysis   - The effect of exercise on walking was examined in six studies (n=546). Four studies reported that exercise significantly improved walking (P<0.05) and two other studies found multicomponent exercise to be no more effective than control intervention (P>0.05). |
| 1. Machado, 2020 | n=6 | Patients with dementia (n=489)  Mean age: 80.9 (from 51 to 93)  Gender: 67% female | Experimental: Multicomponent exercise (30-60 min x 1-2 days/wk x 4-52 wk)  Control: Usual care, social activity, home safety assessment | ADL: BI, IADL, ADCS-ADL, FIM, Katz ADL  Walking: Speed  Balance: SPPB, One-leg balance test | Effect sizes (95% Confidence Interval)  SMD = 0.31 (0.16, 0.46)  No of studies in ES: 11 (n=425)  I^2^ (%) = 8  P<0.01   - The result of the meta-analysis of four studies showed that multicomponent exercises significantly improved ADL. - Two of the three studies in which walking speed was assessed found statistically insignificant changes (n=124; P> 0.05), whilst another study reported a significant increase in walking speed (n=134; P< 0.05). - The review reported that multicomponent exercises had no significant impacts on balance (n=231; P> 0.05). |
| 1. Marques, 2019 | n=2 | Patients with AD (n=207)  Mean age: 65 to 100  Gender: NR | Experimental: Aerobic or anaerobic exercises (30 min x 3-5 days/wk x 26-52 wk)  Control: Usual care, no intervention | ADL: Pfeffer instrumental activities questionnaire | No meta-analysis   - Only one study with very low-quality had data investigating the effect of exercise on ADL. No statistically significant difference was found between the groups (n=2, P> 0.05). |
| 1. Russ, 2020 | n=9 | Patients with dementia (n=456)  Mean age: 85.5  Gender: 74.1% female | Experimental: High-intensity functional exercise (45-60 min x 2-3 days/wk x 12-16 wk)  Control: Usual care, social activities | ADL: BI, FIM  Walking: speed  Balance: BBS | Effect sizes (95% Confidence Interval)  SMD = ADL: 0.30 (0.11, 0.49), MD = Balance: 2.30 (0.44, 4.16)  n (ES) = ADL: 3, Balance: 3  I^2^ (%) = ADL: 0, Balance: 73  P= ADL: 0.002, Balance: 0.02   - It was found that high-intensity functional exercises significantly improved ADL (n=426) and balance (n=417) in individuals with dementia. - For gait speed, one study found significant effects of exercise on gait speed (P=0.034) whereas another found that exercise was not effective for gait speed (P=0.86). |
| 1. Sultana, 2020 | n=5 | Patients with dementia, AD and MCI (n=150)  Mean age: from 65 to 85  Gender: NR | Experimental: Exergames Wii Fit^©^  Time, duration and frequency: Not reported  Control: Usual care, education, walking, exercise | Balance: BBS, TUG | Effect sizes (95% Confidence Interval)  SMD = 0.46 (0.08, 0.84)  No of studies in ES: 4 (n=112)  I^2^ (%) = 0  P=0.02   - Meta-analysis of data from four studies showed that exergames significantly improved balance ability. |
| 1. Van Santen, 2018 | n=3 | Patients with dementia (n=71)  Mean age: 75  Gender: 59% female | Experimental: Exergames (30-60 min x 3-5 days/wk x 7-8 wk)  Control: Walking, no intervention, cognitive training | ADL: ADL, IADL  Walking: TUG  Balance: BBS, Tinetti | No meta-analysis   - Data from all three studies (n=71) showed that although exergames intervention improved ADL more than no intervention (P<0.001), it was not superior to walking and cognitive intervention (P>0.05). - The effect of exergames on mobility was examined in two studies (n=52). Only one study found exergaming more effective in improving balance than control intervention (P<0.001). |
| 1. Wei, 2020 | n=12 | Patients with dementia (n=981)  Mean age: 60 to 85  Gender: NR | Experimental: Tai chi (20-60 min x 2-5 days/wk x 8-26 wk)  Control: Usual care, social activities, health education | ADL: Lawton’s IADL, FAQ  Visuospatial Function: BDT VST | No meta-analysis   - Two studies found that tai chi improved ADL significantly more than the control intervention (n=227; P < 0.05). - Likewise, only two studies assessed visuospatial function (n=327). One reported a more significant improvement in the visuospatial function of the experimental group (P < 0.05), whereas the results of another study showed that the difference between the groups was not significant (P > 0.05). |
| 1. Yang, 2020 | n=11 | Patients with MCI (n=1061)  Mean age: 74.1  Gender: 71% female | Experimental: Tai chi (30-120 min x 1-6 days/wk x 10-52 wk)  Control: Usual care, memory training, education, social activities, stretching exercise, no intervention | Visuospatial function:  RFT, BDT, BCT | Effect sizes (95% Confidence Interval)  SMD = 0.29 (0.10, 0.48)  No of studies in ES: 7 (n=432)  I^2^ (%) = 0  P=0.003   - Meta-analysis of data from three studies showed that tai chi exercise significantly improved visuospatial ability. |
| 1. Yeh, 2021 | n=15 | Patients with dementia (n=860)  Mean age: ≥65  Gender: 72% female | Experimental: High-intensity functional exercise (45-120 min x 2-3 days/wk x 12-16 wk)  Control: Usual care, social activities | ADL: BI  Walking: 4MWT, 6MWT, GAITRite  Balance: BBS, inertial sensor | Effect sizes (95% Confidence Interval)  SMD = ADL: 0.80 (0.23, 1.38), Balance: 0.57 (0.31, 0.83), Gait speed: 0.08 (-0.02, 0.18)  n (ES) = ADL: 3, Balance: 4, Gait speed: 5  I^2^ (%) = ADL: 0, Balance: 54, Gait speed: 75  P= ADL: 0.006, Balance: 0.0001, Gait speed: 0.04   - According to the moderate quality of evidence, it was found that high-intensity functional exercises significantly improved ADL (n=420), gait speed (n=587) and balance (n=524) in individuals with dementia. Moreover, it was reported that this intervention had a long-term positive effect (ADL: P<0.05, gait speed: P=0.0007 and balance: P=0.07). |
| 1. Zhang, 2019 | n=5 | Patients with dementia, AD and MCI (n=803)  Mean age: 65 to 79  Gender: NR | Experimental: Traditional Chinese exercises including tai chi, baduanjin, qigong, Jinjiang, liuzijue and wuqinxi (20-50 min x 3-5 days/wk x 12-52 wk)  Control: Usual care, physical activity, relaxation or stretching exercise | Visuospatial function:  VS, BDT | Effect sizes (95% Confidence Interval)  SMD = 0.38 (0.22, 0.54)  No of studies in ES: 3 (n=660)  I^2^ (%) = 0  P<.001   - In three studies, the effect of traditional Chinese exercises on visuospatial function was investigated. Meta-analysis results showed that exercise significantly improved visuospatial ability. |
| 1. Zhao 2020 | n=10 | Patients with dementia and MCI (n=702)  Mean age: 79.8  Gender: 58% female | Experimental: Exergaming training and virtual reality-based exercises (30 -120 min x 1-5 days/wk x 4-24 wk)  Control: Relaxation and flexibility exercises, walking, no intervention, stretching | ADL: BI, IADL  Balance: BBS, wearable sensors  Visuospatial function: ROCFT | No meta-analysis   - In four studies, the effect of exergaming intervention on balance was examined (n=179). Three studies reported that the intervention significantly improved balance (P < 0.05). - Likewise, three studies evaluated the effect of exergaming on ADL (n=650), and only one study reported a positive result. - In a study in which visuospatial function was evaluated, significant improvement was reported in both groups, but the difference between the two groups was insignificant (n=18; P=0.69). |
| 1. Zheng, 2016 | n=11 | Patients with dementia (n=1497)  Mean age: 74.1  Gender: 62% female | Experimental: Aerobic exercises (30-60 min x 2-5 days/wk x 26-52 wk)  Control: Education, usual physical activity, stretching exercises, social activities | Visuospatial function:  UFOV | No meta-analysis   - No significant improvement was found in visuospatial function (P>0.05). |
| 1. Zhou, 2020 | n=11 | Patients with MCI (n=676)  Mean age: from 68 to 79  Gender: 66% female | Experimental: Multicomponent exercise, resistance exercise, aerobic exercise, high-speed elastic band training, tai chi, yoga (12-52 wk)  Time and frequency: Not reported.  Control: Health education, usual care, no intervention | Visuospatial function:  BDT | Effect sizes (95% Confidence Interval)  SMD = 0.38 (0.03, 0.72)  No of studies in ES: 2 (n=132)  I^2^ (%) = 0  P=0.03   - Data from two studies showed that exercise had a significant effect on improving visuospatial function. |
| 1. Zhu, 2015 | n=23 | Patients with AD (n=886)  Mean age: from 50 to 96  Gender: 66% female | Experimental: Aerobic, tai chi, dance, walking, physical activities (6-52 wk)  Time and frequency: Not reported  Control: NR | ADL: BI, IADL  Balance: BBS  Walking: 6MWT, Speed, TUG | Effect sizes (95% Confidence Interval)  SMD = ADL:0.78 (0.33, 1.23), Balance: 1.11 (0.37, 1.84), 6MWT: 2.23 (-0.47, 4.93), Gait speed: 0.16 (-0.14, 0.47)  n (ES) = ADL: 5, Balance: 5, 6MWT: 3, Gait speed: 6  I^2^ (%) = ADL: 62, Balance: 66, 6MWT: 96, Gait speed: 19  P= ADL: 0.0007, Balance: 0.003, 6MWT: 0.11, Gait speed: 0.29   - The effect of physiotherapy on the performance of ADLs was investigated in five studies (n=272). No significant results were found in one study (P=0.82), but it was reported that ADL improved significantly in other studies. - Likewise, although statistically significant results on balance were reported (n=113), physical exercises were found to have a statistically insignificant effect on walking speed (n=237) and distance (n=128) compared with control groups. |
| 1. Zhu, 2020 | n=16 | Patients with AD (n=1181)  Mean age: 69 to 83  Gender: NR | Experimental: Aerobic, strength, walking, physical activities, flexibility (30-150 min x 1-7 days/wk x 8-48 wk)  Control: Usual care, stretching exercise, social and cognitive activities | ADL: BI, IADL, Physical function of SF36, ADCS-ADL | Effect sizes (95% Confidence Interval)  SMD = 0.68 (0.19, 1.16)  No of studies in ES: 10 (n=515)  I^2^ (%) = 86  P< 0.05   - The results of a meta-analysis of data from ten studies showed that physical activity significantly improved ADL. The results of the regression of covariates showed that age (P=0.47), frequency (P=0.204), time (P=0.72), duration (P=0.492) and intensity (P=0.281) had no significant effect. |
| 1. Zou, 2019 | n=12 | Patients with MCI (n=1298)  Mean age: from 60 to 77.8  Gender: 68% female | Experimental: Tai chi, yoga, qigong (30-90 min x 1-6 days/wk x 12-52 wk)  Control: Health education, usual care, no intervention | Visuospatial function:  BDT, Copy cube/ TMT b | Effect sizes (95% Confidence Interval)  SMD = 0.51 (0.15, 0.87)  No of studies in ES: 2 (n=123)  I^2^ (%) = 0  P=0.01   - Data from two studies showed that mind-body exercises had a significant effect on improving visuospatial function. |

2MWT, 2-minute walk test; 6MWT, 6-minute walk test; 8MWT, 8-minute walk test; 10MWT, 10-minute walk test; ACIF, Acute Care Index of Function; AD, Alzheimer’s disease; ADCS-ADL, Alzheimer’s Disease Cooperative Study Group Activities of Daily Living scale; ADL, activities of daily living; ANT, Animal Naming Test; B-ADL-25, Bayer Activities of Daily Living; BBS, Berg balance scale; BDS, block design score; BI, Barthel Index; CADS, Changes in Advanced Dementia Scale; CDT, Clock-Drawing Test; DAD-ADL, Disability Assessment for Dementia subscale Activities of Daily Living; E-ADL, Erlangen test of Activities of Daily Living; FRT, Functional Reach Test; GS, gait speed; JTT, Jebsen Taylor Hand Function Test; IADL, instrumental activities of daily living; IDDAD, Interview for Deterioration of Daily Activities in Dementia; LBS, Lawton and Brody’s scale; VS, visual span; UPDRS-II, Unified Parkinson’s disease rating scale subscale II (ADL); ROCFT, Rey-Osterrieth Complex Figure Test; SPPB, short physical performance battery; TUG, time and up go; UFOV, useful field of view

1. **AMSTAR-2 scores of reviews**

| **Author** | **1** | **2** | **3** | **4** | **5** | **6** | **7** | **8** | **9** | **10** | **11** | **12** | **13** | **14** | **15** | **16** | **Risk of Bias** |
| --- | --- | --- | --- | --- | --- | --- | --- | --- | --- | --- | --- | --- | --- | --- | --- | --- | --- |
| Almeida 2020 | Y | Y | Y | PY | Y | N | N | Y | Y | N | Y | N | Y | Y | N | Y | CLQ |
| Brett 2016 | Y | N | N | PY | Y | Y | N | Y | Y | N | NM | NM | Y | Y | NM | Y | CLQ |
| Cai 2020 | Y | N | Y | PY | Y | Y | N | PY | Y | N | Y | N | N | N | Y | Y | CLQ |
| Chan 2020 | Y | Y | Y | PY | Y | Y | N | Y | Y | N | Y | Y | Y | Y | Y | Y | LQ |
| Farhang 2019 | Y | N | Y | PY | Y | Y | Y | Y | Y | N | NM | NM | Y | N | NM | Y | LQ |
| Forbes 2015 | Y | Y | Y | Y | Y | Y | Y | Y | Y | N | Y | Y | Y | Y | Y | Y | HQ |
| Groot 2016 | Y | N | N | PY | N | N | N | PY | Y | N | Y | Y | Y | Y | Y | Y | CLQ |
| Karssemeijer 2017 | Y | Y | N | PY | Y | N | N | PY | Y | N | Y | Y | Y | Y | Y | Y | LQ |
| Lam 2018 | Y | N | Y | PY | Y | Y | N | Y | Y | N | Y | Y | Y | Y | Y | Y | CLQ |
| Lee 2016 | Y | N | N | PY | Y | Y | N | PY | N | N | Y | N | N | N | Y | Y | CLQ |
| Leng 2018 | Y | N | Y | PY | Y | Y | N | PY | Y | N | Y | N | N | Y | N | Y | CLQ |
| Lewis 2017 | Y | N | Y | PY | Y | Y | N | Y | Y | N | Y | Y | Y | Y | Y | Y | CLQ |
| Li 2019 | Y | N | N | PY | Y | Y | N | Y | Y | N | Y | Y | Y | Y | Y | Y | CLQ |
| Lim 2019 | Y | N | N | PY | Y | N | N | Y | Y | N | NM | NM | N | Y | NM | Y | CLQ |
| Long 2019 | Y | Y | N | PY | Y | Y | N | Y | Y | N | NM | NM | Y | Y | NM | Y | LQ |
| Machado 2020 | Y | Y | N | PY | Y | N | N | Y | Y | N | PY | N | N | Y | Y | Y | CLQ |
| Marques 2019 | Y | N | N | PY | Y | Y | PY | Y | Y | N | Y | Y | Y | N | N | Y | CLQ |
| Russ 2020 | Y | N | N | PY | Y | Y | N | Y | Y | N | Y | Y | Y | Y | N | Y | CLQ |
| Sultana 2020 | Y | N | Y | PY | Y | Y | N | Y | Y | N | Y | Y | Y | Y | N | Y | CLQ |
| Van Santen 2018 | Y | Y | N | PY | Y | Y | N | Y | Y | N | NM | NM | Y | N | NM | N | LQ |
| Wei 2020 | Y | Y | N | PY | N | Y | N | Y | Y | N | N | N | Y | Y | N | Y | CLQ |
| Yang 2020 | Y | N | Y | Y | Y | Y | N | Y | Y | N | Y | Y | Y | Y | N | Y | CLQ |
| Yeh 2021 | Y | Y | N | PY | Y | Y | N | Y | Y | N | N | Y | Y | Y | N | Y | CLQ |
| Zhang 2019 | Y | N | N | PY | Y | Y | N | Y | Y | N | Y | N | Y | Y | N | Y | CLQ |
| Zhao 2020 | Y | Y | N | PY | Y | Y | N | Y | Y | N | NM | NM | Y | Y | NM | Y | LQ |
| Zheng 2016 | Y | Y | Y | Y | N | Y | N | Y | Y | N | Y | N | Y | Y | Y | Y | LQ |
| Zhou 2020 | Y | Y | N | Y | Y | Y | N | Y | Y | N | Y | Y | Y | Y | N | Y | LQ |
| Zhu 2015 | Y | N | N | PY | N | Y | N | Y | Y | N | PY | N | N | Y | Y | Y | CLQ |
| Zhu 2020 | Y | N | N | PY | Y | N | N | Y | Y | N | Y | Y | Y | Y | Y | Y | CLQ |
| Zou 2019 | Y | N | Y | PY | Y | Y | N | Y | Y | N | PY | N | Y | N | Y | Y | CLQ |

CLQ: Critically low quality; LQ: Low quality; HQ: High quality; Y: Yes; N: No; PY: Partial Yes; NM: No meta-analysis conducted

AMSTAR 2 Criteria:

1. Did the research questions and inclusion criteria for the review include the components of PICO?
2. Did the report of the review contain an explicit statement that the review methods were established prior to the conduct of the review and did the report justify any significant deviations from the protocol?
3. Did the review authors explain their selection of the study designs for inclusion in the review?
4. Did the review authors use a comprehensive literature search strategy?
5. Did the review authors perform study selection in duplicate?
6. Did the review authors perform data extraction in duplicate?
7. Did the review authors provide a list of excluded studies and justify the exclusions?
8. Did the review authors describe the included studies in adequate detail?
9. Did the review authors use a satisfactory technique for assessing the risk of bias (RoB) in individual studies that were included in the review?
10. Did the review authors report on the sources of funding for the studies included in the review?
11. If meta-analysis was performed did the review authors use appropriate methods for statistical combination of results?
12. If meta-analysis was performed, did the review authors assess the potential impact of RoB in individual studies on the results of the meta-analysis or other evidence synthesis?
13. Did the review authors account for RoB in individual studies when interpreting/ discussing the results of the review?
14. Did the review authors provide a satisfactory explanation for, and discussion of, any heterogeneity observed in the results of the review?
15. If they performed quantitative synthesis did the review authors carry out an adequate investigation of publication bias (small study bias) and discuss its likely impact on the results of the review?
16. Did the review authors report any potential sources of conflict of interest, including any funding they received for conducting the review
